# Assessment of the Topology and Oligomerisation States of Coiled Coils Using Metadynamics with Conformational Restraints

**DOI:** 10.1021/acs.jctc.4c01695

**Published:** 2025-03-05

**Authors:** Evangelia Notari, Christopher W. Wood, Julien Michel

**Affiliations:** † EaStCHEM School of Chemistry, University of Edinburgh, David Brewster Road, Edinburgh EH9 3FJ, U.K.; ‡ School of Biological Sciences, University of Edinburgh, Roger Land Building, Edinburgh EH9 3FF, U.K.

## Abstract

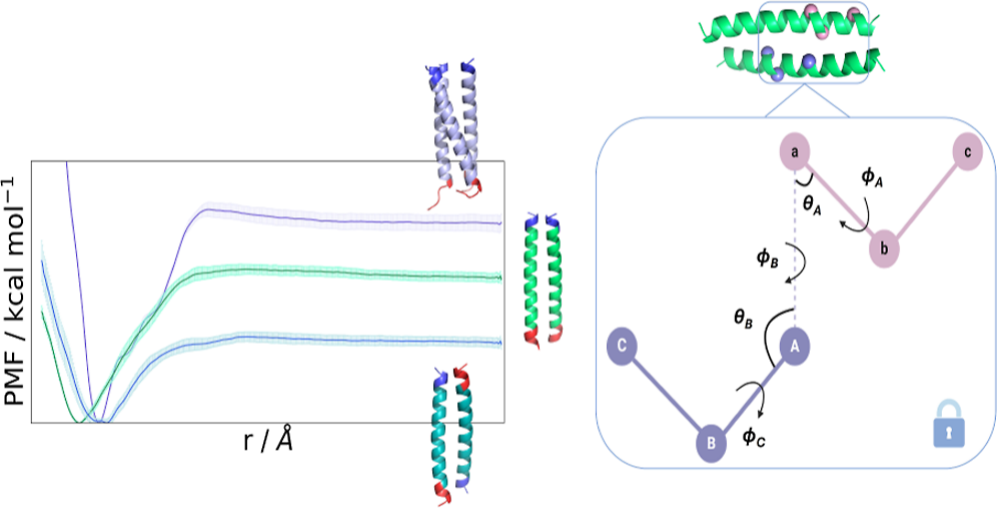

Coiled-coil proteins provide an excellent scaffold for multistate *de novo* protein design due to their established sequence-to-structure relationships and ability to switch conformations in response to external stimuli, such as changes in pH or temperature. However, the computational design of multistate coiled-coil protein assemblies is challenging, as it requires accurate estimates of the free energy differences between multiple alternative coiled-coil conformations. Here, we demonstrate how this challenge can be tackled using metadynamics simulations with orientational, positional and conformational restraints. We show that, even for subtle sequence variations, our protocol can predict the preferred topology of coiled-coil dimers and trimers, the preferred oligomerization states of coiled-coil dimers, trimers, and tetramers, as well as the switching behavior of a pH-dependent multistate system. Our approach provides a method for predicting the stability of coiled-coil designs and offers a new framework for computing binding free energies in protein–protein and multiprotein complexes.

## Introduction

**Figure 1:**
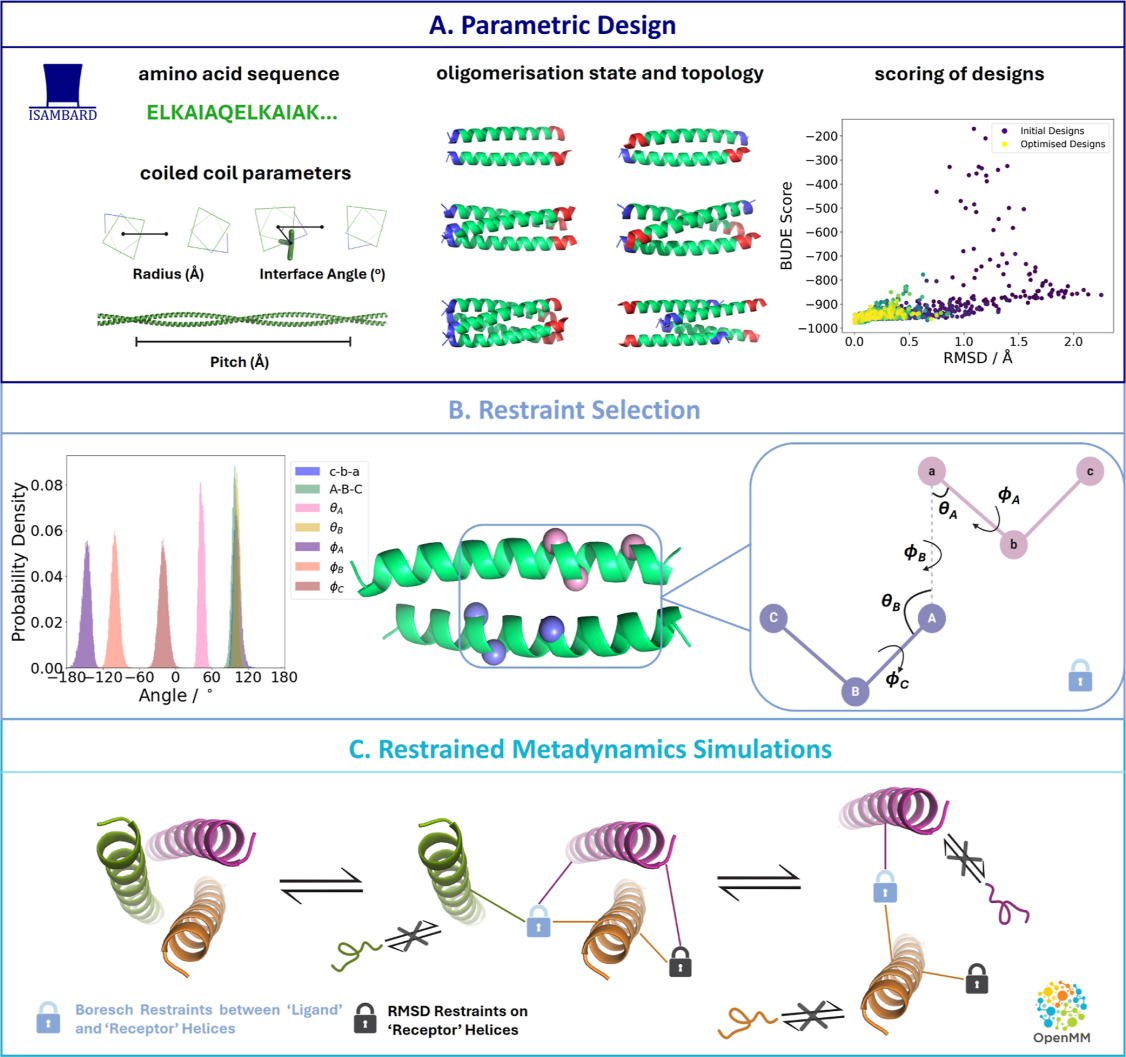
Proposed pipeline for the calculation of binding free energies of *de novo* coiled coils. (A) Parametric design of coiled coils in ISAMBARD, with user-specified amino acid sequence, topology and oligomerization state. The radius, interface angle and pitch are optimized during design. The designed structures are scored with the BUDE force field. Each design is compared to the design with the lowest BUDE score by their RMSD. (B) Selection of anchor points for Boresch restraints. Three Cα atoms are chosen on each of the “ligand” and “receptor” moieties. Angle and torsion distributions are obtained from equilibrium simulations of the complex. (C) Metadynamics simulations are carried out sequentially in OpenMM, with one helix being removed from the assembly at a time. Torsional restraints are applied on each monomer helix to prevent unfolding upon dissociation. The orientation and position of the “ligand” relative to the “receptor” is restrained with Boresch restraints, and the “receptor” is additionally restrained with RMSD restraints to prevent its rearrangement upon dissociation of the “ligand”.

Coiled coils are bundles of two or more α-helices that wrap around each other to form superhelical complexes.^1^ Coiled coils can adopt a variety of parallel, antiparallel or mixed (e.g., up–down–up) topologies, which can be characterized with three geometric descriptors: the coiled coil radius, interface (Crick) angle and pitch (Figure 1A).^2^ The assemblies possess a distinctive type of packing termed knobs-into-holes (KIH), where a side chain from one helix (“knob”) packs into a diamond-shaped cavity (“hole”) formed by four side chains in a different helix of the bundle.^3^ This distinguishes coiled coils from other helical bundles, which are characterized by less intimate packing.^4,5^ This packing arrangement is supported by sequence repeats of hydrophobic (*h*) and polar (*p*) residues,^3^ usually in heptad repeats *hpphppp*, often denoted in the form of an *abcdefg* register. In these heptad repeats, the hydrophobic residues have an average spacing of 3.5 residues, giving rise to amphipathic helices, composed of a hydrophobic and a hydrophilic face. Since there is a small spacing deviation between 3.5 in heptad repeats and 3.6 in ideal α-helices, the hydrophobic face of a helix with 3.5 spacing proceeds around the helix, leading to supercoiling of helices upon assembly rather than packing in a straight manner.^6^ Coiled-coil assembly is driven by the hydrophobic effect, where the helices assemble to bury their hydrophobic faces in a hydrophobic core.^7^ The nature of the hydrophobic residues at the *a* and *d* positions of the heptad repeat largely determines the oligomerization state of coiled-coil dimers, trimers and tetramers.^8,9^ Further helices can be recruited to the assembly via the expansion of the hydrophobic interface, achieved by introducing hydrophobic residues at the *e* and *g* positions.^10^

The wealth of knowledge about these topological arrangements have contributed to turning coiled coils into an attractive scaffold for *de novo* computational protein design. However, the majority of *de novo* designed coiled coils are hyperstable, with melting points exceeding 100 °C.^11,12^ Natural coiled coils, on the other hand, are dynamic and play central roles in a multitude of biological functions.^13−17^ As a consequence, attention has shifted to the *de novo* design of switchable coiled coils. While this has been accomplished in a number of cases,^18−31^ achieving the desired balance between multiple target coiled-coil states often requires several rounds of trial-and-error and a detailed understanding of the energetic factors that discriminate between alternative topologies and oligomerization states. For these reasons, achieving quantitative predictions of coiled-coil stability by calculating the free energy of oligomerization has been deemed one of the outstanding challenges in coiled coil research.^32^ Such capability would pave the way for the computational design of switchable proteins for a variety of biotechnological applications.

The free energy landscape of coiled coils is complex, consisting of multiple local, energetically close minima.^32,33^ Currently, little experimental free energy data are available for *de novo* designed coiled coils, due to their high thermostability. Initial attempts on predicting oligomerization states in coiled coils focused on distinguishing dimers from trimers.^34−39^ Further studies expanded predictions to higher order oligomerization states (up to pentamers) and alternative topologies.^40−42^ More recently, deep learning methods have been employed to distinguish dimers, trimers and tetramers.^43−45^ However, the ability of the aforementioned approaches to predict the stability of higher order oligomerization states has been limited by the dearth of available experimental structural data for model training and calibration. Furthermore, these approaches do not account for external stimuli during the prediction of oligomerization state (e.g., change in the pH or temperature, or addition of ligands), and are consequently not suitable for multistate coiled-coil design.

Methods such as MM-PBSA and MM-GBSA,^46,47^ which are based on molecular dynamics (MD) simulations, offer an alternative route for computing the binding free energies of protein–protein systems^48−50^ including coiled-coil complexes.^42,51,52^ However, when applied to protein assemblies, these methods frequently produce binding free estimates with large statistical errors^53^ and make a number of approximations, such as neglecting or partially accounting for the effects of changes in internal energy and entropy on protein association free energies, resulting in limited accuracy.^54^ More rigorous free energy methods such as alchemical binding free energy calculations have become established for the computation of binding free energies of small molecules to proteins with MD.^55−60^ While in theory an alchemical pathway could be constructed via decoupling a binding partner in a protein–protein complex from its environment in the bound and unbound states, the alchemical decoupling of a complete protein molecule will usually result in poor convergence^61^ due to large free energy changes.^62^ This is expected to be particularly problematic for coiled coils, given the magnitude of electrostatic interactions present due to their multiple charged residues.

Alternatively, a so-called path-based approach may be adopted to compute protein–protein binding free energies with MD methods. In this approach, the potential of mean force (PMF) for dissociating a protein assembly can be computed via a variety of enhanced sampling techniques applied to preselected collective variables (CVs).^62^ While the path-based PMF approach appears more tractable, it is still difficult to obtain converged free energy estimates for large biomolecules with complex geometries.^53,62−68^ More precise free energy changes can be obtained through the application of restraints which ensure that the relative orientation and position of the ligand with respect to the receptor is preserved and therefore the available phase space to be sampled during the simulations is reduced.^54,63−65^ To this end, Woo and Roux pioneered the so-called geometrical route for binding free energy calculations, where the binding process is decomposed into distinct, independent processes, each described by a unique CV, which corresponds to a specific slow degree of freedom of the system. In the original implementation of the geometrical route,^54^ three atom centers are chosen on both binding counterparts, constructing a local frame of reference. The orientation of the ligand with respect to the receptor is then defined by the three Euler angles Θ, Φ, Ψ, and the position of the ligand with respect to the receptor is defined by the polar angles θ and ϕ. The ligand is additionally restrained in the average conformation it adopts when bound to its receptor with the application of an RMSD restraint. The binding free energy is then quantified by means of a series of sequential PMF calculations on the above CVs. In each PMF calculation, the only restraints present are those that have been added beforehand. The final PMF calculation involves the physical separation of the two binding partners along the Euclidean distance *r* between their centers of mass and is carried out in the presence of all restraints. Simulations without such restraints have been reported to fail to converge and to yield binding free energies inconsistent with experimental data.^62,65,69^ The geometrical route has been automated with quaternion-based angle restraints^67^ as CVs in the NAMD^70,71^ and GROMACS^72^ MD engines. Additional variations of the framework have also been introduced, with various implementations of RMSD restraints applied on both receptor and ligand in the bound and unbound states.^62,62,65,69^ The protocol has so far been successfully used in calculating the binding free energy of a variety of protein–protein complexes, in good agreement with experimental data,^54,62,64,65,67,73−76^ including a homodimeric α-helical complex.^69^

An alternative set of orientational and positional restraints has been proposed by Boresch et al.^77^ in the context of small molecule-protein binding free energy calculations. Boresch restraints are constructed by selecting three anchor points (atoms or groups of atoms) on the receptor and three on the ligand. The restraints correspond to the external degrees of freedom of the ligand, and consist of a distance *r*, two angles θ_*A*_, θ_*B*_ and three dihedrals ϕ_*A*_, ϕ_*B*_, ϕ_*C*_. The Boresch angles and dihedrals play the same role as the Euler and polar angles used in the geometrical route, but are easier to implement in other MD software, such as OpenMM.^78^ Numerous techniques having been developed to facilitate the automated selection of anchor points.^79−85^ To the best of our knowledge, Boresch restraints have not been used so far in binding free energy calculations via a geometrical route. We decided to employ the Boresch angles and dihedrals instead of the Euler and polar angles, since no quaternion implementation is available in OpenMM to date. In principle, the binding free energy should be independent of the type of restraints, the atom centers selected for the construction of the restraints, or the values of the force constants,^64,77,86^ provided stable restraints that have been appropriately selected.^87^

**Figure 2:**
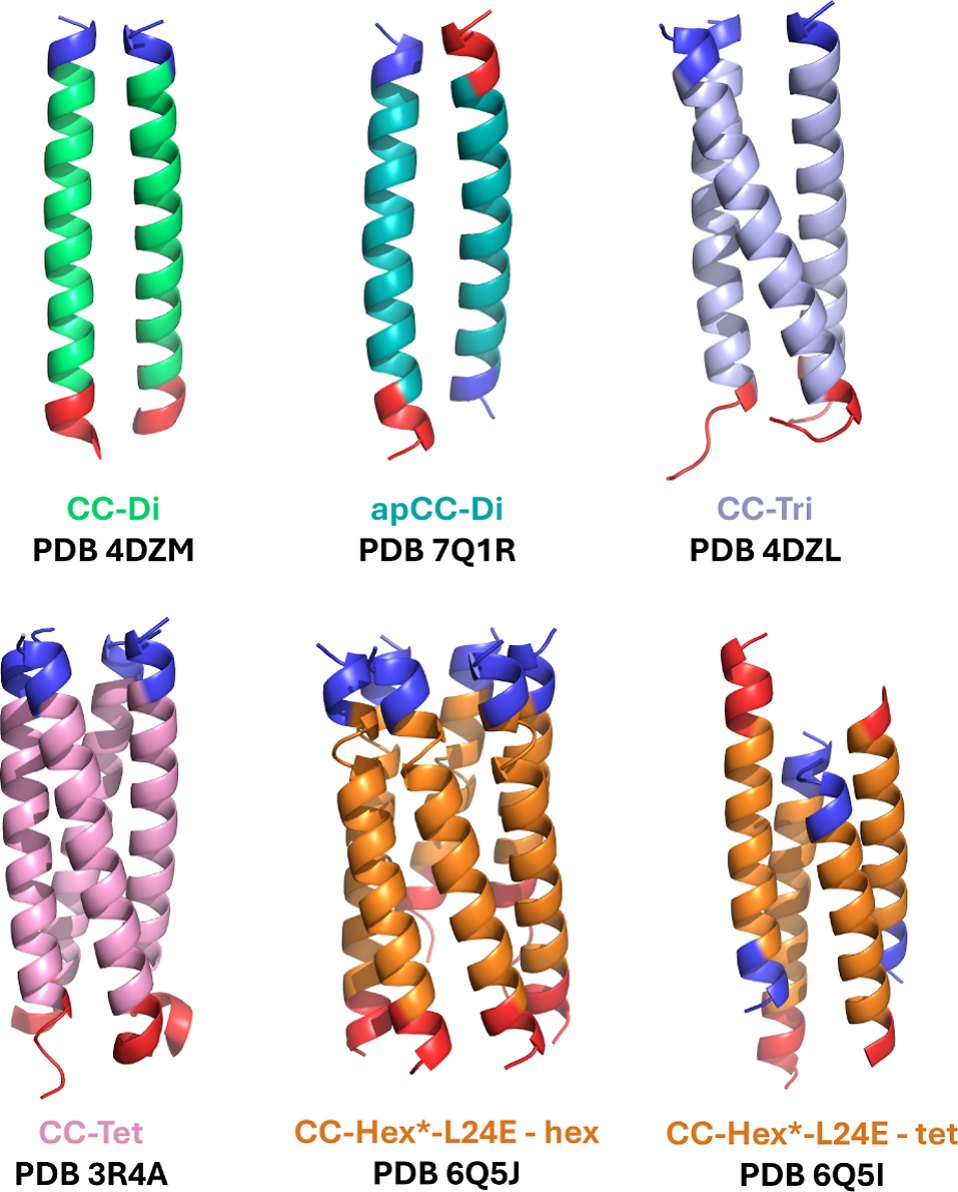
*De novo* coiled coils studied and their PDB accession codes. Blue denotes N-terminus and red denotes C-terminus. Rendered with PyMOL.^91^

In the present work, we combine well-tempered metadynamics (WT-metaD)^88^ with Boresch restraints^77^ in OpenMM^78^ to explore the oligomerization and topology preferences of *de novo* designed coiled coils. The overall pipeline is summarized in Figure 1. We demonstrate the applicability of our protocol by predicting: the topology (parallel vs antiparallel) preferences of CC-Di, CC-Tri and apCC-Di, the oligomerization state preferences of CC-Di, CC-Tri and CC-Tet, and the pH switching behavior of the CC-Hex*-L24E system, which forms a parallel hexamer at low pH and an antiparallel tetramer at high pH (Figure 2).^29,33,89^ We also show that employing WT-metaD^88^ and funnel metadynamics^90^ in the absence of orientational and positional restraints fails to yield reliable free energy estimates. Overall our results demonstrate the potential of path-based MD methods to act as a late stage filter in multistate protein design.

## Theory

We provide the theoretical framework for using Boresch restraints in binding free energy calculations via a geometrical route, and rederive the Boresch standard state correction term in the context of the geometrical route.

Consider a solution in thermodynamic equilibrium, composed of receptor and ligand molecules that can associate to form a receptor (R)–ligand (L) complex in a bimolecular fashion. The equilibrium binding constant *K*_eq_ of the receptor–ligand association
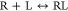
is defined in terms of the concentrations of each species [R], [L] and [RL] as *K*_eq_ = . The standard free energy of the receptor–ligand binding, Δ*G*_b_^◦^, can be expressed as
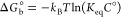
1where *k*_B_ is the Boltzmann constant, *T* is the absolute temperature and *C*° is the standard concentration of 1 M, equal to 1/*V*° = 1/1661 Å^3^, where *V*° is the standard state volume.

According to the geometrical route framework proposed by Woo and Roux,^54^ the binding constant *K*_eq_ can be expressed as a function of the free energy contributions from the applied conformational (c), orientational (o) and positional (a) restraints in the bound (site) and unbound (bulk) states (eq 2).
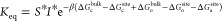
2where β = . S* is a surface area term that corresponds to the fraction of a sphere of radius r* that is centered on the binding site of the receptor and is accessible to the ligand (eq 3), and describes the removal of the positional restraints on the ligand when it is dissociated from the receptor.^62,65^
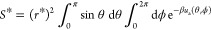
3where *u*_a_ is the sum of the restraint potentials applied on the polar angles θ and ϕ, and r* is a point in the bulk, far from the receptor binding site. S* is typically evaluated numerically. I* is a one-dimensional integral over the distance *r* between the centers of mass of the binding partners, and is defined in terms of the separation PMF *W*(*r*), calculated in the presence of all the restraints (eq 4).^54,65^
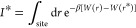
4The free energy terms Δ*G*_c_^site^, Δ*G*_o_^site^, Δ*G*_a_^site^ and Δ*G*_c_^bulk^ in eq 2 can be evaluated in sequential, one-dimensional PMF calculations.^92^ The term Δ*G*_o_^bulk^ can be calculated analytically as an angular integral (eq 5) and corresponds to the orientational movement of an unbound partner in the bulk.^65,67^
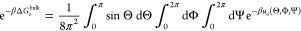
5where *u*_o_ is the sum of the restraint potentials applied on the Euler angles Θ, Φ and Ψ.

Taking into account eqs 2–5, eq 1 can be expanded and rearranged as follows
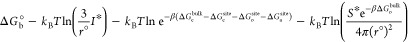
6where the standard state volume term has been divided and distributed across terms in eq 6, in order to keep the terms in the logarithms unitless: . The term 4π(*r*°)^2^ can be thought of as the “standard state surface area” *A*°, corresponding to the surface area of a sphere of volume *V*°. The term *r*° =  can be thought of as the “standard state distance”, corresponding to the radius of a sphere of volume *V*°.

The first two terms in eq 6 represent the free energy contributions from the individual PMF calculations (separation, conformational, orientational and positional restraints in the bound state, and conformational restraints in the unbound state). The last term in eq 6 is a correction that accounts for the release of the ligand to the standard state surface area after dissociation from the receptor; it corrects for the release of all angular restraints in the bulk solvent
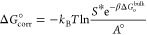
7

Instead of using the Euler and polar angles to describe the orientation and position of the ligand relative to the receptor, one could instead use the degrees of freedom proposed by Boresch et al.^77^ In this case, the integral S* (eq 3) at a distance r* where the binding partners do not interact can be rewritten^93^ as
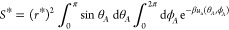
8where θ_*A*_ and ϕ_*A*_ are the angle and dihedral angle between anchor points A, a, b, c (Figure 1B).

Equally, the orientational integral  can be rewritten^93^ as
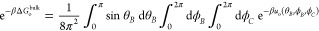
9where θ_*B*_, ϕ_*B*_, ϕ_*C*_ are the angle and dihedral angles between anchor points a, b, A, B, C (Figure 1B).

If harmonic restraints are employed to restrain the Boresch angles and dihedrals to their equilibrium values, then eqs 8 and 9 become
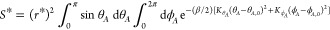
10
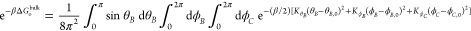
11where *K* is a force constant and 0 denotes the value of the respective degree of freedom at equilibrium. Equations 10 and 11 can be further simplified to eqs 13 and 14 if the Gaussian integral (eq 12) is used^77^
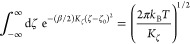
12
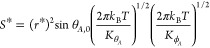
13
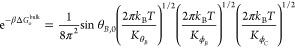
14where it is assumed that the integrals in eqs 10 and 11 can be extended to ±∞, and that sin θ_A_ and sin θ_B_ can be treated as the constants sin θ_A,0_ and sin θ_B,0_, and moved out of the integrals. Both assumptions are strongly justified for appropriately selected restraints which are not subject to instabilities.^87^

Taking into account eqs 13 and 14, the angular correction free energy of eq 7 can be expressed as
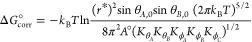
15The full standard state correction can be recovered from the combination of eq 15 and the first term in eq 6, which account for releasing the ligand to the standard state area and standard state distance, respectively.

Equation 6 can generally quantify the free energy of a ligand binding to a receptor, provided appropriate restraints are employed. Since the coiled coils in the present study are all homomultimers composed of identical α-helical monomers, the effect of symmetry must also be considered during the calculation of the binding free energy.^63,69^ Therefore, a symmetry correction must be applied to the binding free energy as follows
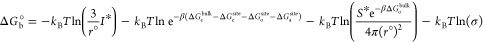
16where σ is a symmetry factor. It has been shown that σ =  in the case of a homodimer.^63,69^ It can be further shown that for a homomultimeric coiled coil of oligomerization state *n*, the symmetry factor σ is . A detailed derivation is provided in the Supporting Information (Section S1).

## Methods

### Decoupling Oligomerization into Folding and Association

In our protocol, we decouple the coiled coil oligomerization process into folding and association. While in nature folding and association of coiled coils occur simultaneously,^94^ they can be studied separately *in silico* due to free energy being a state function. In our protocol, WT-metaD simulations with Boresch restraints are ran on fully folded α-helices, and the cost of folding each helix is implicitly accounted for by dividing the association free energy by the number of helices in the assembly, similar to approaches followed by Ramos et al. and Rämisch et al.^40,42^ For oligomerization states higher than dimers, the association is further decomposed into separate steps, with one α-helix being removed from the assembly in each step, and the total free energy of association of the coiled-coil assembly is calculated by summing up the contributions from the individual association steps (Figure 1).

### Structure Preparation

The structures of CC-Di, apCC-Di, CC-Tri, CC-Tet and CC-Hex*-L24E in both its tetrameric and hexameric state were obtained from the PDBe (Figure 2 and Table S1).^95^ For CC-Di, CC-Tri and CC-Tet, any C-terminal residues missing from the X-ray structures were rebuilt with PyMOL,^91^ and the 4-iodo-phenylalanine at position 22 in CC-Di and CC-Tri was mutated to tryptophan as per the original peptide sequence. No C-terminal missing residues were rebuilt in the CC-Hex*-L24E structures, as the number of residues missing (7 residues missing from chains C and D in the tetramer, and 6 residues missing from chain E in the hexamer) implied high flexibility of these regions, and imposing a predefined conformation would not be realistic. All peptides were additionally capped with acetyl and amide groups at the N- and C-termini of each helix respectively in accordance with the experimental structures.

The remaining structures (competing topologies and oligomerization states) were designed in ISAMBARD, employing parametric modeling.^96^ The competing topology structures include an antiparallel variant of CC-Di, a parallel variant of apCC-Di and an up–down–up variant of CC-Tri. The competing oligomerization state structures include CC-Di designed as a parallel trimer and tetramer, CC-Tri designed as a parallel dimer and tetramer, and CC-Tet designed as a parallel dimer and trimer. CC-Di was also designed as a parallel dimer, to compare with the simulations of the X-ray structure (Figure S1) and to investigate whether using experimental or designed structures affects the outcome of the free energy calculations. For each design, the sequence, register, topology and oligomerization state were fixed, with the radius, interface angle and pitch allowed to vary. In the case of antiparallel assemblies, an additional parameter was included to modulate the offset between the helices. Designs were chosen based on their BUDE scores and the convergence of the design process.^97^ The chosen designs were also capped with acetyl and amide groups at the N- and C-termini of each helix, respectively. Further details about the design process and parameters can be found in the Supporting Information (Section S2) and at michellab/CCmetaD.

The modeling of the CC-Hex*-L24E system at different pH conditions was done by assigning fixed protonation states to the Glu residues; at low pH, all Glu residues were protonated, whereas at high pH all the Glu residues were deprotonated. Experimental data suggest CC-Hex*-L24E forms a parallel hexamer at pH 2–6.5 and an antiparallel tetramer at pH > 7.^29^

### Choice of Force Field and Implicit Solvent Model

We chose to carry out the free energy calculations in implicit solvent, since our aim was to develop a protocol that can be used for rapid calculations of binding free energies of coiled coils. Currently, the computational cost of explicit solvent simulations prevents their routine use in protein design pipelines.^98^ The use of implicit solvent models is therefore highly desirable for protein design purposes, due to its high computational efficiency and faster conformational sampling compared to explicit solvent.^99,100^ However, implicit solvent models have been deemed insufficient for accurately reproducing the secondary structure preferences of peptides,^101−103^ particularly peptides with a high content of charged residues,^98,104^ due to their tendency to produce salt-bridge-trap conformations.^101−108^ Nevertheless, while coiled coils have a considerable number of native salt bridges, which may be mistreated with an implicit solvent model, the main driving force for coiled coil assembly is the hydrophobic effect,^7^ which can be adequately modeled with implicit solvent. Additionally, the use of conformational, orientational and positional restraints during the binding free energy calculations precludes the formation of non-native salt bridges.

We carried out a preliminary study to select a suitable force field and generalized Born implicit solvent model for preserving the helicity of coiled-coil monomers when unbound (see Supporting Information Section S3 for details). The ff96/OBC2 parameter sets^109,110^ were found to be the most adequate and were used for all subsequent metadynamics simulations and free energy calculations.

### Well-Tempered Metadynamics Simulations

WT-metaD simulations were run with the metadynamics class of OpenMM 7.5.^78,88^ The simulations were run at 298.15 K employing a Langevin thermostat with a damping coefficient of 1 ps^–1^. Hydrogen mass repartitioning was used, with the mass of each hydrogen atom set to 1.5 amu. The integration time step was 4 fs. It was also deemed necessary to introduce additional restraints on the ϕ, ψ torsions of coiled-coil monomers to further preserve their helicity. Three protocols were considered in this study. Additional details about the simulation protocols are given in the Supporting Information (Sections S4 and S5).

#### Unrestrained Metadynamics

Initial WT-metaD simulations of the coiled coil dimers CC-Di and apCC-Di were run in the presence of only the helical ϕ, ψ restraints for 1 μs with three independent replicates, with the same starting conformation but different starting velocities.

#### Funnel Metadynamics

Funnel metadynamics simulations^90^ were explored as a means to improve sampling and reproducibility among replicates. A custom funnel design was implemented in OpenMM inspired by the approach followed by Hedges et al.^111^ (Figure S6). Funnel metadynamics simulations of CC-Di, apCC-Di and CC-Tri were run for 1 μs with three independent replicates, with the same starting conformation but different starting velocities. A range of different funnel parameters were tested on each complex. Further details about the setup of funnel metadynamics simulations are provided in the Supporting Information (Section S4).

#### Metadynamics with Boresch Restraints

The modified geometrical route with the Boresch restraints was employed to calculate the binding free energies of the coiled coils depicted in Figure 2. When we refer to binding free energies Δ*G*_bind_^◦^ hereafter, we mean association free energies of fully folded α-helices. Coiled coils of different oligomerization state are then compared with the metric Δ*G*_bind_^◦^ per helix (divided by the number of helices in the complex). Details about the metadynamics protocol with Boresch restraints are provided in the Supporting Information (Section S5).

## Results and Discussion

### Unrestrained Metadynamics and Funnel Metadynamics Simulations Fail to Accurately Describe Coiled-Coil Association

Preliminary metadynamics simulations of the coiled-coil dimers CC-Di and apCC-Di in the absence of restraints (with the exception of the helical ϕ, ψ restraints) yielded similar results to those reported by Blazhynska et al.^65,69^ when running unrestrained PMF calculations for protein–protein complexes, namely limited reproducibility among replicates (Figure S5). This can be partly attributed to the increased number of available conformations of the separated coiled-coil monomers in the bulk solvent. We therefore investigated whether funnel metadynamics^90^ could yield better reproducibility via restricting the bulk space available to the monomers upon dissociation. While improved reproducibility was achieved in certain cases, this was found to be largely dependent on the shape of the funnel, and differed between coiled-coil assemblies (Figure S7). Inspection of the bound states from the simulations revealed further complications. While sometimes reasonable bound states could be obtained (Figure S7A), in the majority of simulations unreasonable bound conformations prevailed (Figure S7B–D). Analysis of interhelical contacts from the bound state conformations showed a wide variety of contacts formed, which are not present in the native coiled-coil dimers (Figures S8 and S9). Interestingly, hotspots of interactions appear approximately every seven residues, which can be explained by the repetitive nature of coiled coils. The monomer sequences of CC-Di and apCC-Di consist of 4 heptad repeats each; it is plausible that when the monomers attempt to reassociate, heptad *i* of one helix can form interactions with heptad *i* – 1 or *i* + 1 from the other helix, resulting in the partial stabilization of misfolded structures (Figure S7B,D). This is further exacerbated by the formation of non-native salt bridges, a common caveat of implicit solvent. For example, Glu2 in helix A in CC-Di forms non-native salt bridges with Lys14 and Lys21 from helix B, whereas the native salt bridge with Lys7 is not formed (Figure S8). Similar salt bridge traps can be seen in apCC-Di, e.g. Glu4-Arg20, Glu4-Arg21 and Glu6-Arg21 (Figure S9). This renders the obtained PMF profiles unreliable, as the minima do not correspond to the desired bound states. In fact, multiple local minima can be observed, corresponding to different misfolded structures with non-native contacts (Figure S7).

Further complications arose when running funnel metadynamics simulations of the coiled-coil trimer CC-Tri. Similarly to the funnel metadynamics simulations of CC-Di and apCC-Di, the representative bound states did not resemble the desired native coiled-coil structures (Figure S10). This is supported by inspecting the interhelical interactions formed between helix A (“ligand”) and helices B and C (“receptor”), where multiple undesired contacts can be seen (Figure S12). Additionally, the interhelical interactions between helices B and C revealed that the “receptor” is rearranging in the absence of the “ligand”, forming a coiled-coil dimer, with salt bridges on both sides of the complex. This is in contrast with the salt bridges expected on the “receptor”, which should form only on one face of the complex (Figure S11). This introduced two further complications to the calculations. First, helix A can not dock properly to a rearranged “receptor” BC. Second, the dimer separation PMF of the “receptor” complex BC invalidates the free energy pathway, as the “receptor” has been allowed to adopt a “closed” conformation with additional interhelical interactions, resembling a dimeric coiled coil (Figure S12C,D).

Consequently, the lack of reproducibility, the misfolded bound states and the rearrangements of the “receptor” complexes obtained from the unrestrained and funnel metadynamics prompted us to employ additional conformational, orientational and positional restraints.

### Restrained Metadynamics Simulations Can Predict the Favored Topology of Coiled-Coil Dimers and Trimers

We initially tested our restrained metadynamics protocol on whether we could predict the preferred topology (parallel vs antiparallel) of the coiled-coil dimers CC-Di and apCC-Di. Running simulations of alternative topologies is not possible in the absence of orientational and positional restraints, where the monomer helices are free to tumble upside down when dissociated, and can reassociate with potentially different topologies.

**Figure 3:**
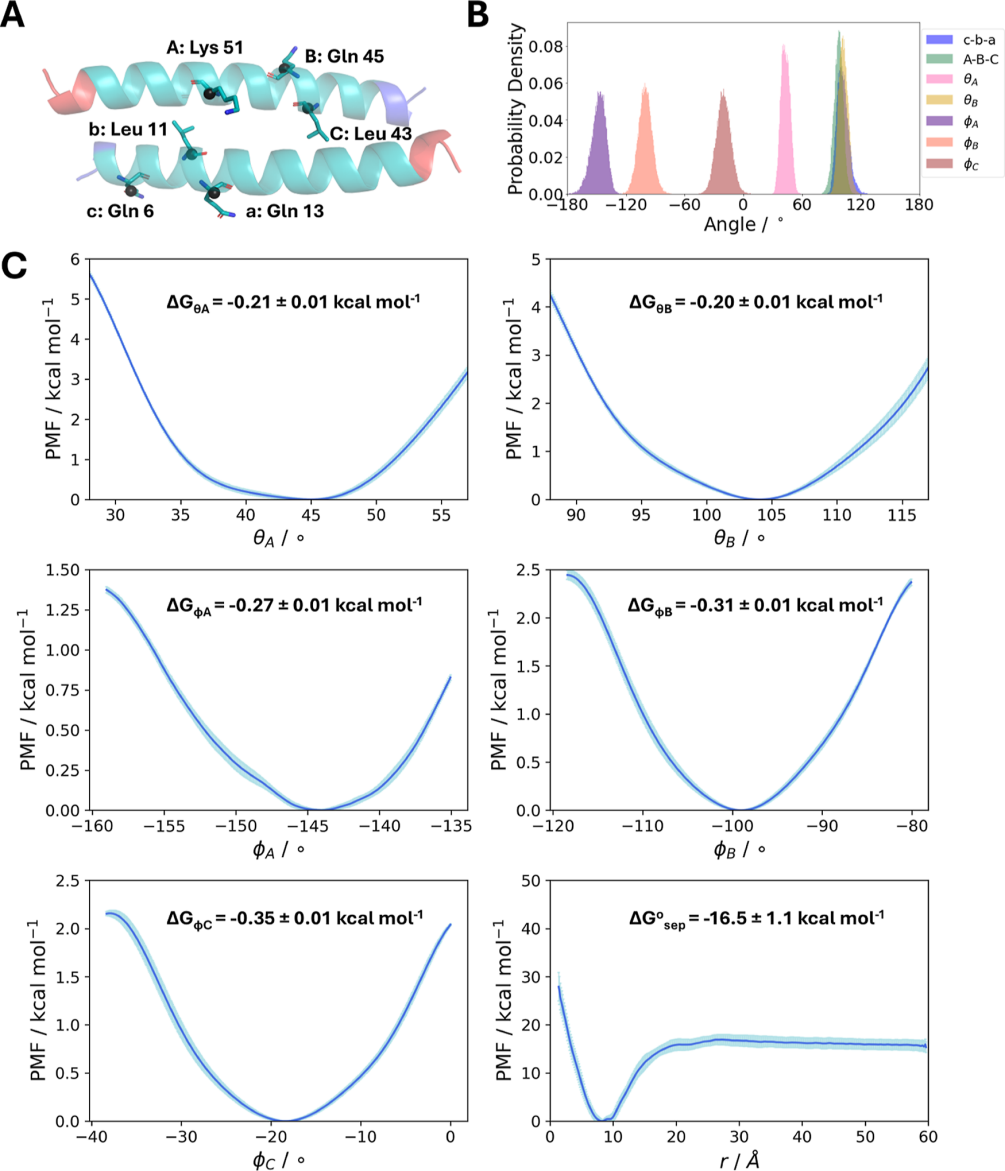
Boresch restraints selection and PMF profiles for the apCC-Di dimer. (A) The selected anchor point residues are depicted as sticks. The Boresch restraints are imposed on the Cα atom of each residue, shown as a black sphere. The N-termini of the peptide are shown in blue and the C-termini in red. Rendered with PyMOL.^91^ (B) The distributions of the Boresch angles and dihedrals and the two unrestrained angles *c*–*b*–*a* and *A*–*B*–*C*. All angles should be away from 0° or 180°. (C) The PMF profiles of each CV, obtained from running simulations with the modified geometrical route.

**Figure 4:**
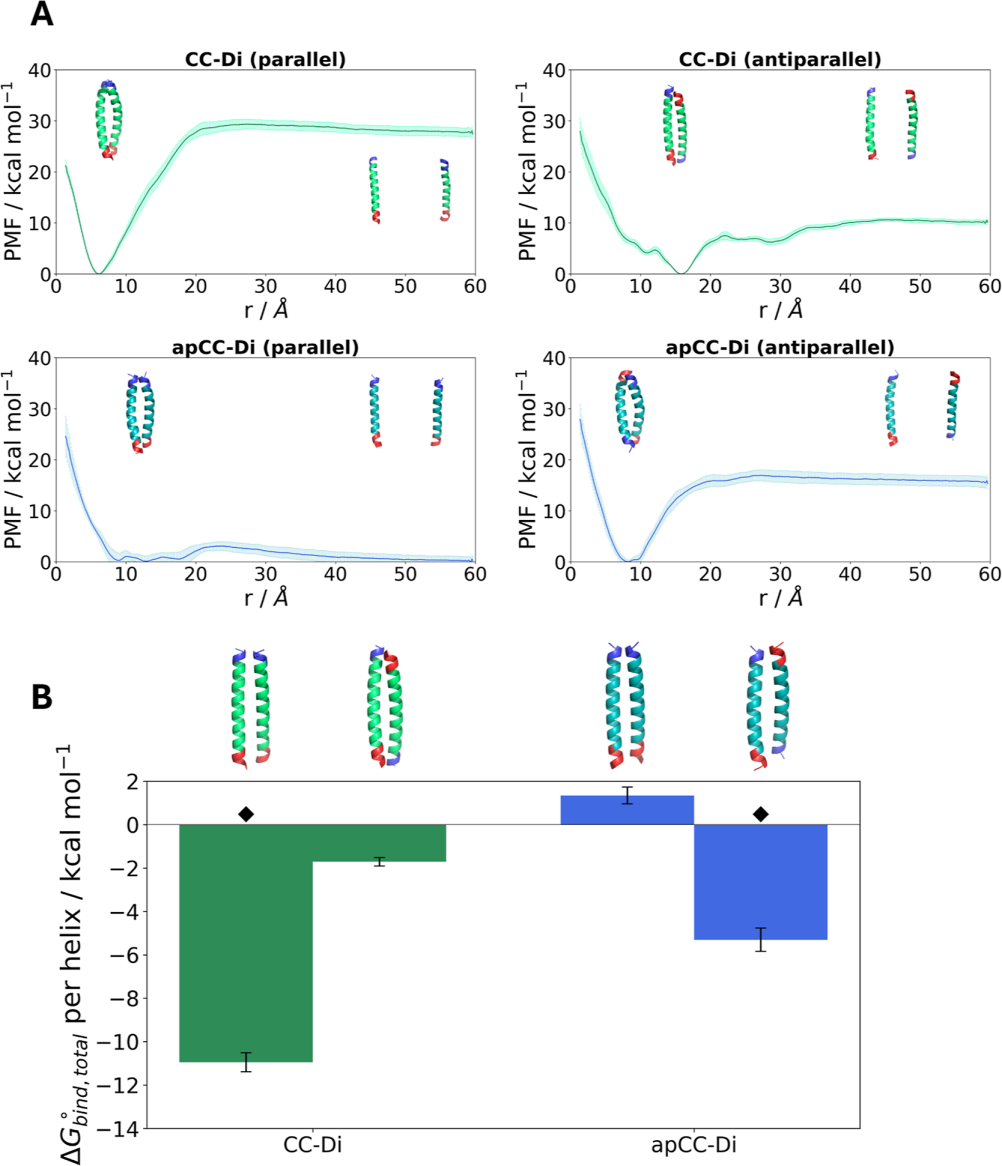
(A) Separation PMF profiles for CC-Di and apCC-Di in their parallel and antiparallel conformations. Representative structures of the bound and unbound states are shown for each case. The shaded areas represent standard error. The N-termini are shown in blue and the C-termini are shown in red. (B) The total binding free energy for each dimer, divided by the number of helices in the assembly. The bars represent standard error. ⧫ denotes the native structures. For comparison of dimers, it is not necessary to divide by the number of helices in the assembly, but this has been done here to facilitate comparisons with higher oligomerization states.

The free energies from each CV, along with the Boresch correction and the symmetry correction, are reported in Table S2, and representative PMFs are shown in Figures 3 and S15 for apCC-Di and CC-Di, respectively. The restrained metadynamics protocol correctly predicts the preferred topology for each dimer; CC-Di preferably forms a parallel dimer, and apCC-Di preferably forms an antiparallel dimer. Similar to the original geometrical route framework, the angle and dihedral PMFs contribute less than 1 kcal mol^–1^ each, which are small but not negligible contributions to the total binding free energy. The largest contribution is attributed to the separation PMF, as expected, reflecting the intermolecular interactions formed between “receptor” and “ligand” upon binding.^54^ Crucial differences between the parallel and antiparallel assemblies can already be observed from the separation PMF profiles (Figure 4A). While the depth of the separation PMF does not correspond to the binding free energy due to the presence of restraints, it can be clearly seen that the parallel CC-Di and the antiparallel apCC-Di are the preferred structures. The separation PMFs have clear, distinct global minima (Figure 4A), with the exception of the parallel apCC-Di complex, which shows multiple minima in the 10–20 Å range. Isolation of the bound conformations from the separation PMF minima showed that reasonable structures were obtained. Analysis of interhelical interactions showed that the contacts from the native coiled-coil structures were rescued (Figure S16). Of notable importance are the Asn17-Asn17 hydrogen bond in CC-Di, and the Asp@e-Arg@a hydrogen bonds/salt bridges in apCC-Di, which determine the formation of parallel and antiparallel dimers, respectively.^33,89^ These interactions are observed in the restrained metadynamics simulations, whereas they are not as prominent in the unrestrained and funnel metadynamics simulations (Figures S8 and S9).

We additionally ran Boresch-restrained metadynamics simulations of a structure of CC-Di designed in ISAMBARD, to compare to the simulations of the X-ray structure. The same anchor points and force constants for the Boresch restraints were used. Equal binding free energies were obtained for the two structures within error (Table S3), showing that our protocol performs equally well on experimental and designed structures, provided the designed structures are reliable. This comparison is especially significant for CC-Di, since its asymmetric unit in the X-ray structure does not contain the dimer interface.^33^ For the remainder of our simulations, we used experimental structures where they were available, and designed any alternative states with ISAMBARD. We also investigated whether using an alternative set of anchor points would yield a comparable binding free energy in the case of CC-Di. Again, equal binding free energies were obtained within error (Table S3), showcasing the protocol’s independence from the anchor points chosen, provided they have been chosen to avoid numerical instabilities.

**Figure 5:**
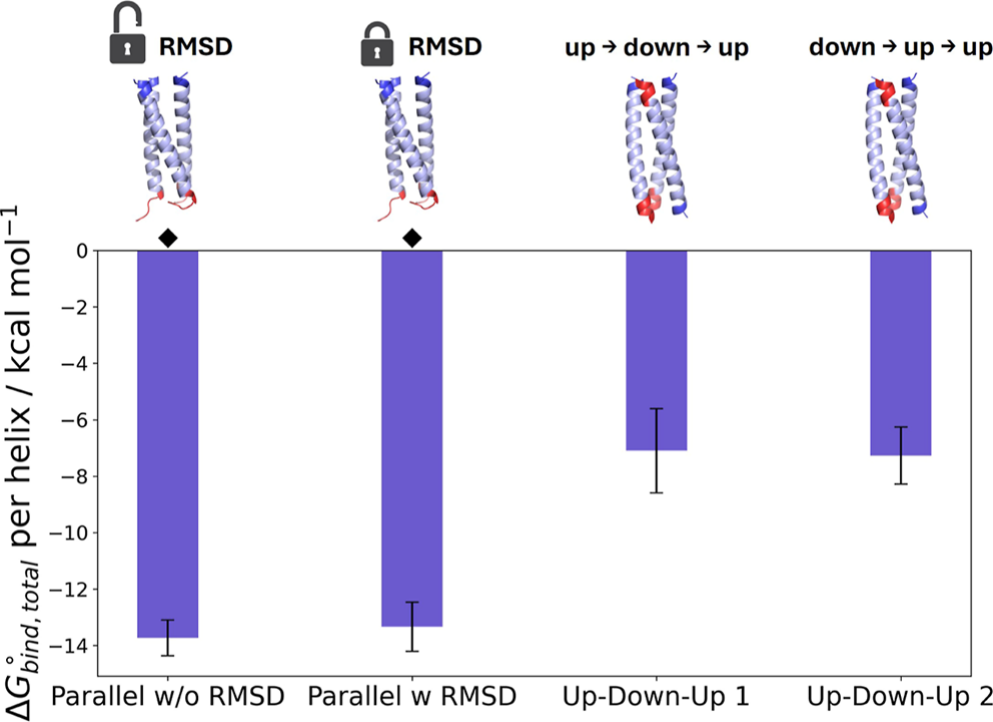
Total binding free energy for the parallel and up–down–up CC-Tri coiled coils, divided by the number of helices in the assembly. Simulations of the parallel CC-Tri were carried out both in the absence and presence of RMSD restraints on the “receptor” helices. Simulations of the up–down–up CC-Tri were carried out with two different separation schemes, with a different order of helices separated from the assembly in each case. The bars represent standard error. ⧫ denotes the native structures. For comparison of trimers, it is not necessary to divide by the number of helices in the assembly, but this has been done here to facilitate comparisons with other oligomerization states.

We then employed our protocol to assess whether we can predict the preferred topology of a higher order coiled coil, separating one helix from the assembly at a time. To this end, the coiled-coil trimer CC-Tri was simulated as an all-parallel trimer (native structure) and as an up–down–up variant (competing structure). Interestingly, the parallel CC-Tri was the only higher order assembly whose “receptor” (helices B–C) could reorganize from a “closed” to an “open” conformation during the trimer separation simulations, and did not necessitate the use of RMSD restraints on the receptor helices, although this did not happen reproducibly. Notably, this was not possible in the funnel metadynamics simulations in the absence of orientational and positional restraints. We carried out two sets of simulations for CC-Tri, one with and one without RMSD restraints on the receptor helices (Tables S4, S5 and Figures S17–S19). This served as a control for the additional RMSD restraints, as the binding free energy should be the same after correcting for the presence of restraints, regardless of the type and number of restraints used. When the contribution of the RMSD restraints in the bound and unbound states is accounted for, the binding free energy for the trimer step is consistent with that from the simulations without the RMSD restraints (Figure 5 and Table S4). In order to obtain the total binding free energy for CC-Tri, the contributions from the trimer and dimer steps are taken into account (Table S5). Isolation of the bound conformations from the trimer step separation PMF minima and contact analysis reveal that the desired contact profile is maintained (Figures S11 and S20), with salt bridges formed on only one side of the complex. The same analysis carried out on the dimer step also reveals that the restraints are effective in preserving the intermediate dimer complex in its “open” conformation (Figure S20D).

Simulations of the competing up–down–up variant of CC-Tri served two purposes: to assess whether the correct trimer topology can be predicted, and to verify that the order by which the helices are separated from the assembly does not affect the total binding free energy of the complex. While the individual free energies from each step are expected to be different depending on the helix being removed, the total binding free energy should be the same, given that free energy is a state function. To this end, the up–down–up trimer was separated with two different schemes (Figure S4). The total binding free energies from the two schemes were consistent with each other (Figure 5, Tables S6 and S7), demonstrating that the protocol does not depend on the order in which the monomers are removed from the complex. Additionally, the up–down–up CC-Tri is predicted to be less favorable than the all parallel CC-Tri, establishing that our framework is able to predict the preferred topology of a higher order coiled coil. Interestingly, the Δ*G*_bind_^◦^ of the dimer step from the parallel CC-Tri simulations (−16.2 ± 1.2 kcal mol^–1^, Table S5) is consistent, within error, with the Δ*G*_bind_^◦^ of the dimer step from the up–down–up CC-Tri simulations with scheme 2 (−15.8 ± 2.0 kcal mol^–1^, Table S7), which is in agreement with the fact that these two intermediate dimer complexes are structurally similar, as both have an up–up topology and are in an “open” conformation.

### Restrained Metadynamics Simulations Can Recover the Preferred Oligomerisation State of Coiled-Coil Dimers, Trimers and Tetramers

After establishing that the Boresch-restrained metadynamics simulations are able to predict the preferred topology of coiled coils, we investigated whether our protocol can predict the preferred oligomerization state of the CC-Di, CC-Tri and CC-Tet coiled coils.^33^ Each peptide was modeled as a dimer, trimer and tetramer, and binding free energy calculations were run for each complex (note that the binding free energies for the dimeric CC-Di and trimeric CC-Tri are those computed above).

**Figure 6:**
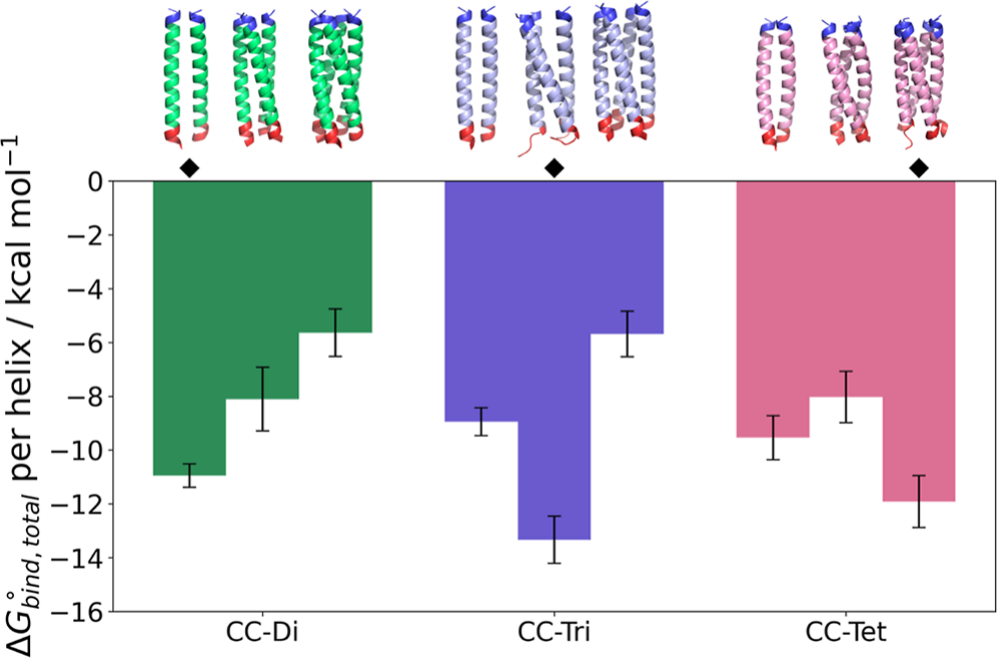
Total binding free energy for each dimeric, trimeric and tetrameric complex of the CC-Di, CC-Tri and CC-Tet sequences, divided by the number of helices in the assembly to allow comparisons between different oligomerization states. The bars represent standard error. ⧫ denotes the native structures.

The obtained binding free energies Δ*G*_bind,total_^◦^/helix are in agreement with the experimental structures, namely CC-Di preferably forms a dimer, CC-Tri preferably forms a trimer and CC-Tet preferably forms a tetramer (Figure 6, Tables S2, S5 and S8–S14). Similar to the original geometrical route framework, the largest contributions to the binding free energy stem from the separation and RMSD PMFs, with their uncertainties also contributing the most to the reported standard error.

**Figure 7:**
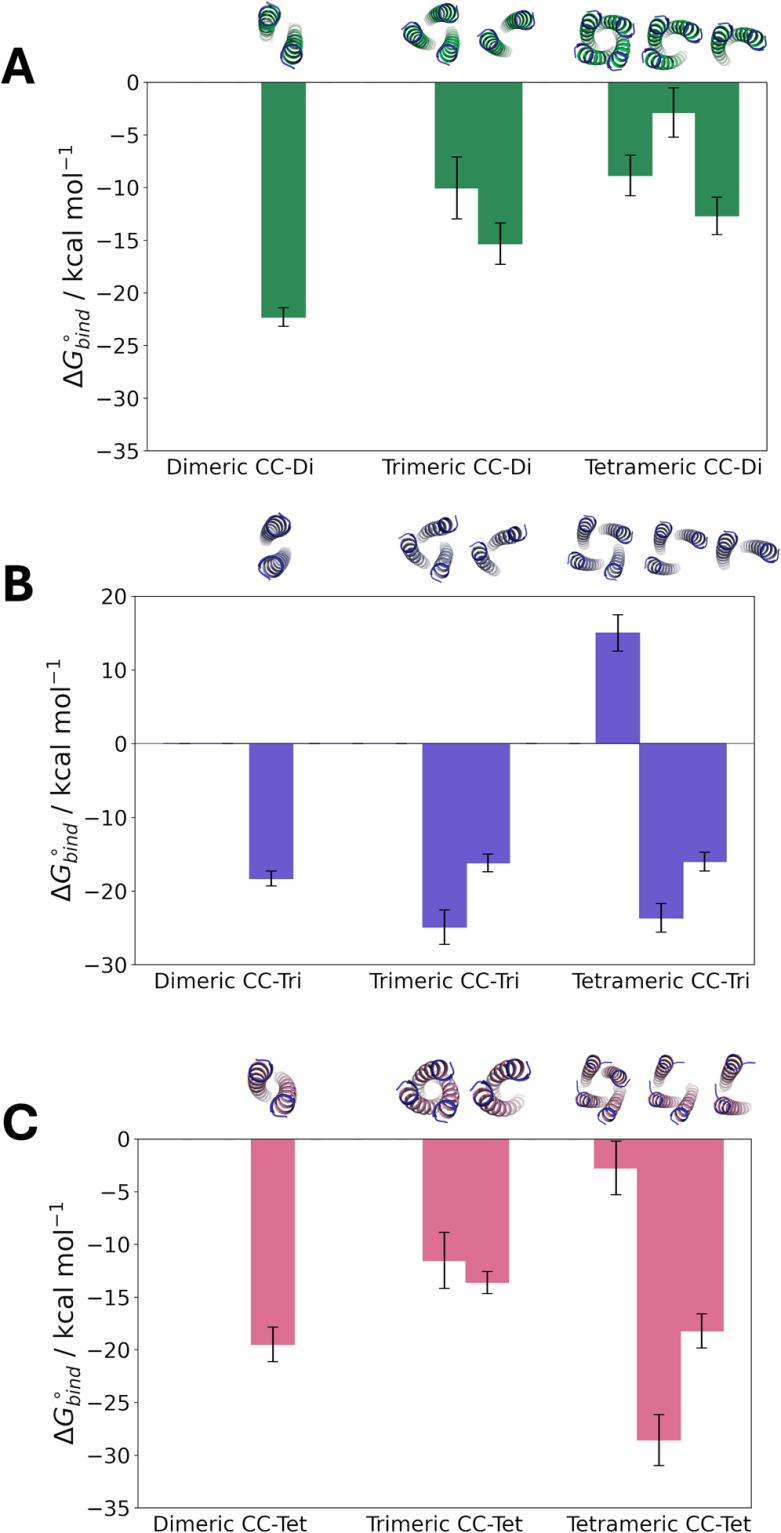
Individual Δ*G*_bind_^◦^ values from each intermediate state calculation for (A) CC-Di, (B) CC-Tri and (C) CC-Tet, each simulated as a parallel dimer, trimer and tetramer. The structures above each Δ*G*_bind_^◦^ value depict the complex being dissociated at each free energy calculation step. The bars represent standard error.

Examination of the Δ*G*_bind_^◦^ of each intermediate state (Figure 7) reveals which helix dissociation event contributes the most to the total binding free energy of the complex. In the case of CC-Di, the dimer dissociation event contributes the most when CC-Di is modeled as a trimer and tetramer. Additionally, it can be seen that the binding free energy for the native dimeric CC-Di is more favorable compared to the binding free energy for the intermediate dimer states in the trimeric and tetrameric CC-Di complexes (Tables S3, S8 and S9). These intermediate dimer states have been restrained in their “open” conformations and therefore lack multiple intermolecular interactions present in the native dimeric CC-Di, which renders their constituting monomers more prone to dissociation. In the case of CC-Tri, the trimer dissociation step contributes the most when CC-Tri is modeled as a trimer and tetramer. In the case of CC-Tet, the trimer dissociation step has the highest contribution. The breakdown of the contributions of each PMF to the binding free energy (Table S14) reveals that while both separation free energies in the tetramer and trimer steps are negative, it is the contribution of the RMSD PMFs in the bound and unbound states  and  that distinguishes the two dissociation steps. By definition  < 0 and  > 0, so the term  quantifies the loss of internal conformational freedom.^54^ This term is highly positive in the case of the tetramer, but less pronounced in the case of the trimer. This overall difference makes the trimer dissociation step appear more favorable. Nevertheless, CC-Tet is still predicted to preferably form a tetramer.

In general, the sign and magnitude of the RMSD difference between the bound and unbound states  indicates whether the rearrangement of the “receptor” helices is favorable or not. A positive RMSD difference indicates that the “receptor” helices are allowed to rearrange more in the unbound state where a helix has been removed, compared to the bound state. On the contrary, a negative RMSD difference indicates that the “receptor” helices are allowed to rearrange more in the bound state compared to the unbound state. This can explain the RMSD differences observed in the three dissociation steps of CC-Tet. In the tetramer step, any rearrangement of the “receptor” helices is limited in the bound state due to the presence of all four helices in a “closed” conformation, and the RMSD difference is therefore highly positive. In the trimer step, the absence of one helix and the “open” conformation of the “receptor” confers more rearrangement freedom to the “receptor” helices in the bound state; the RMSD difference in this case is still positive, albeit less positive compared to the tetramer step. In the dimer step, the RMSD difference is negative, indicating that the intermediate dimer can rearrange more in the bound state than in the unbound state. This is an interesting observation, as one might expect a single α-helix in the bulk to have more conformational freedom than a dimeric complex, especially one that adopts an “open” conformation; however, the ϕ, ψ torsional restraints applied throughout the free energy calculations limit the conformations available to the monomeric α-helix in the bulk. In general, all intermediate dimer dissociation steps have small absolute  values, indicating that there are small differences in the “receptor” reorganization potential between the bound and unbound states.

A similar observation regarding the contribution of the RMSD free energies can be made in the case of the tetrameric CC-Tri, with the tetramer step having a positive Δ*G*_bind_^◦^. Again, the separation PMF is negative (Table S11), whereas the term  is highly positive, rendering the overall dissociation of a single helix from the tetrameric CC-Tri favorable. The “receptor” in this case is composed of three helices with the sequence of CC-Tri, and the positive sign of the  term indicates the preferential rearrangement of the helices in the unbound state, where they can form the native trimeric CC-Tri structure, compared to the bound state, where they are confined by the presence of a fourth helix.

The differences between the native state and alternative competing states can generally be attributed to the amino acid composition of each sequence (Table S1). In the case of CC-Di, each monomer has an Asn residue at the *a* position of one of its central heptads, which strongly favors the parallel dimer state due to the formation of interhelical Asn–Asn hydrogen bonds. Notably, substitution of Asn to Ile, which is ordinarily present at the *a* position in coiled-coil dimers (and is present in all other *a* positions of the CC-Di sequence), results in a parallel trimer instead;^33^ therefore, the presence of the Asn residues confers high selectivity for the parallel dimer state. It has been proposed that, in the dimer state, the cost of burying two Asn residues at the hydrophobic peptide core is offset by the formation of the intermolecular hydrogen bond. However, the burial of more than two Asn residues is not tolerated in higher oligomerization states, rendering the trimer and tetramer states less favorable.^33,112^ The more Asn residues are attempted to be buried at the hydrophobic core, the less stable the assembly, hence the Δ*G*_bind,total_^◦^/helix for the tetrameric CC-Di being less favorable than that of the trimeric CC-Di (Figure 6).

These results demonstrate that our protocol correctly predicts the preferred oligomerization states of CC-Di, CC-Tri and CC-Tet. The differences between their Δ*G*_bind,total_^◦^/helix and those of the alternative competing states suggest a complex free energy landscape, where a few mutations at the hydrophobic core can profoundly alter the preferred oligomerization states.

### Restrained Metadynamics Simulations Capture pH Switching Effects in a *De Novo* Designed Coiled Coil

We then turned our attention to a pH-switchable system, CC-Hex*-L24E, which forms a parallel hexamer at pH 2–6.5 and an antiparallel tetramer with an offset at pH > 7 (Figure 2).^29^ The L24E mutation in the parent hexamer CC-Hex* introduces an acidic residue at one of the *a* positions at the hydrophobic core of the assembly. This is tolerated at low pH values, where the Glu residues are more likely to be protonated and can form a hydrogen bond network further stabilizing the core. However, at higher pH values, the Glu residues are more likely to be deprotonated and negatively charged, and the subsequent repulsion in the otherwise hydrophobic core results in the destabilization of the hexamer and a large structural rearrangement, which leads to formation of the antiparallel tetramer. This is an inherently more challenging system, since the two structurally distinct states must be close in the free energy landscape to allow switching upon changes in the pH.^113^

**Figure 8:**
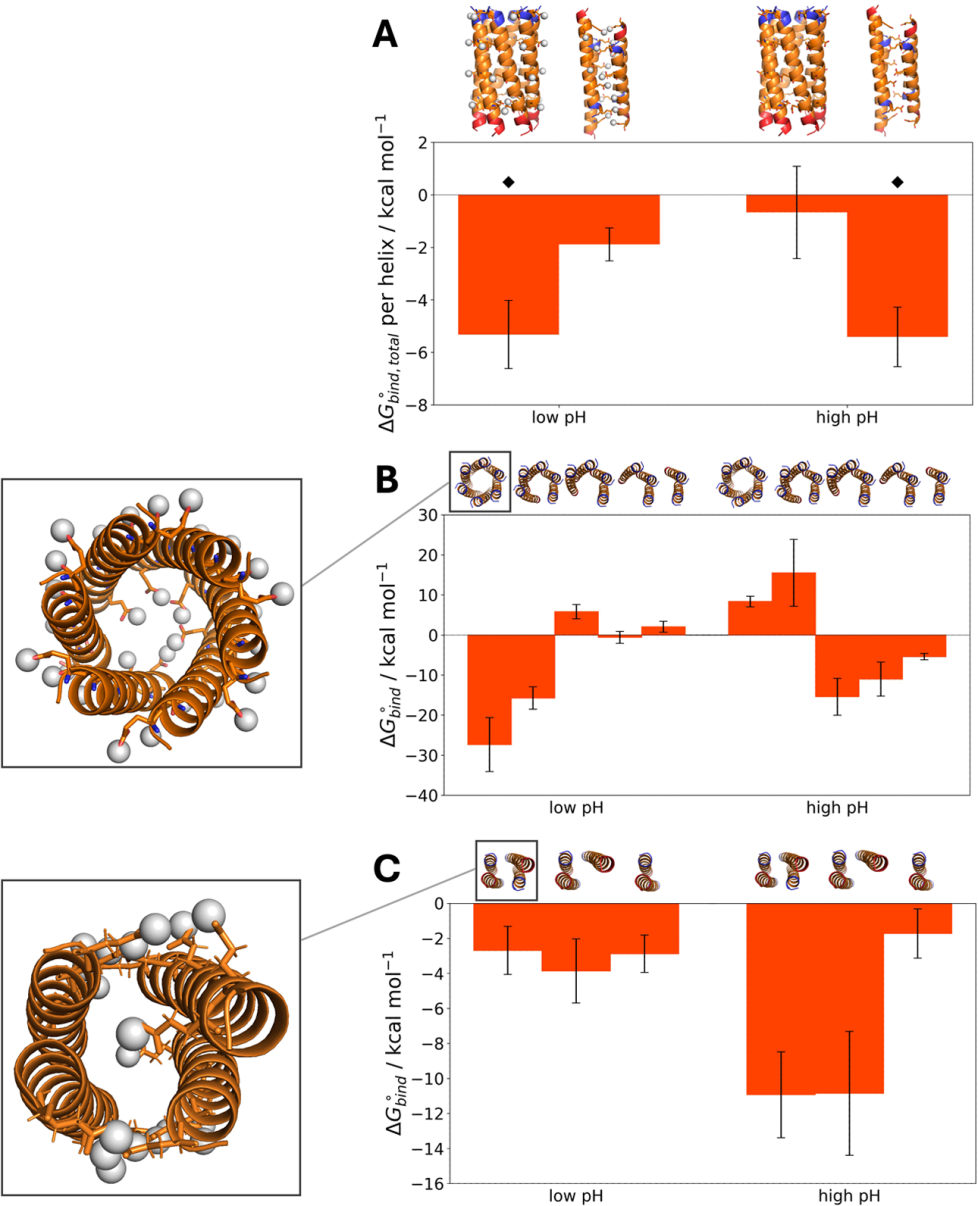
(A) The total binding free energies of the CC-Hex*-L24E hexamer and tetramer at low and high pH conditions, divided by the number of helices in the assembly. The gray spheres represent the hydrogens of the protonated Glu residues at the low pH condition. ⧫ denotes the native structures. (B,C) Individual Δ*G*_bind_^◦^ values from each intermediate state calculation for (B) the parallel hexamer and (C) the antiparallel tetramer. The structures above each Δ*G*_bind_^◦^ value depict the complex being dissociated at each free energy calculation step. The insets provide views from the N- to the C-terminus of the peptides and show the protonated Glu at the core (hydrogens depicted in gray spheres). The bars represent standard error.

The obtained Δ*G*_bind,total_^◦^/helix values correlate with the experimentally observed states, namely the parallel hexamer is more favorable at low pH, and the antiparallel tetramer is more favorable at high pH (Figure 8A and Tables S15–S18). Additionally, the Δ*G*_bind,total_^◦^/helix value of the hexamer at low pH (−5.3 ± 1.3 kcal mol^–1^, Table S17) is consistent with that of the tetramer at high pH (−5.4 ± 1.1 kcal mol^–1^, Table S16), which is in agreement with the experimentally determined switching behavior of the complex. As before, the cost of folding each helix has been implicitly accounted for by dividing the Δ*G*_bind,total_^◦^ with the number of helices in the complex. This folding cost will likely change as the pH changes; indeed, calculations of the percentage of helicity of the CC-Hex*-L24E monomer with Agadir^114−118^ show 13% helicity at pH 2 and 9% helicity at pH 8.5, with intermediate helicities in the pH range 2–8.5. This implies that there will be a higher cost of folding each monomer at high pH, potentially rendering the binding free energies at high pH less favorable. These monomer helicity calculations agree with experimental data which portray the CC-Hex*-L24E complex as highly helical at pH 3–5 and less helical at pH 6–7.^29^ However, this will likely not change the results significantly, as a small difference in monomer helicity is not expected to dramatically change the energetic cost of folding between the low and high pH conditions.

Inspection of the Δ*G*_bind_^◦^ of the intermediate states in the parallel hexamer simulations again reveals which dissociation event contributes the most to the total binding free energy (Figure 8B). An interesting trend emerges for the pH-switchable system: at low pH, the Δ*G*_bind_^◦^ of the hexamer and pentamer dissociation steps are negative, whereas the Δ*G*_bind_^◦^ from the tetramer dissociation event onward are positive, or close to 0 in the case of the trimer, indicating that the tetramer, trimer and dimer intermediate states are unfavorable. On the contrary, at high pH the Δ*G*_bind_^◦^ of the hexamer and pentamer states are positive, and the Δ*G*_bind_^◦^ from the tetramer state onward are negative, indicating that the hexamer and pentamer states are unfavorable. This implies that the tetramer state is a tipping point of the CC-Hex*-L24E system. Even though this intermediate “open” parallel tetramer is structurally different than the antiparallel tetramer experimentally observed at high pH, the change of sign of Δ*G*_bind_^◦^ could provide hints about the presence of a pH-switchable system.

Turning to the antiparallel tetramer simulations, the Δ*G*_bind_^◦^ contributions (Figure 8C) reveal that at low pH each helix separation step contributes to a similar small extent to the Δ*G*_bind_^◦^/helix, with no intermediate state appearing as more favorable. At high pH, equal Δ*G*_bind_^◦^ values from the tetramer and trimer dissociation events primarily contribute to the Δ*G*_bind_^◦^/helix, with the contribution from the dimer intermediate state being close to that in the low pH simulations. Interestingly, the Δ*G*_sep_^◦^ of each dissociation event is equal, within error, at low and high pH (Tables S15 and S16). This implies that interactions of similar type and magnitude are disrupted in each helix dissociation step. The intermolecular interactions primarily affected by changes in the pH and the subsequent changes in the protonation states of titratable residues are salt bridges and hydrogen bonds. The CC-Hex*-L24E antiparallel tetramer however does not have salt bridges or hydrogen bonds in its structure. It has two distinct interface types, which are termed “wide” and “narrow”. These interfaces are flanked by pairs of the same residue from neighboring helices, either Lys or Glu.^29^ As a consequence, the tetrameric CC-Hex*-L24E has alternating seams of positive and negative charge (or lack of charge when the tetramer is modeled at low pH), where no salt bridges can be formed (Figure S23). This explains why the Δ*G*_sep_^◦^ of each dissociation event is equal at low and high pH. It is the RMSD PMFs that differentiate between the low and high pH simulations, with the most striking difference in the  term observed in the tetramer dissociation step (Tables S15 and S16); the highest  at low pH indicates reduced stability of the structure at low pH.

Inspection of the Δ*G*_sep_^◦^ of the CC-Hex*-L24E hexamer reveals additional interesting details (Tables S17 and S18). The Δ*G*_sep_^◦^ of the hexamer, tetramer and dimer dissociation steps are different between the low and high pH conditions, whereas the those of the pentamer and trimer steps are equal, within error. In the hexamer step, the more negative Δ*G*_sep_^◦^ at low pH could be attributed to the initial disruption of the hydrogen bond network formed by the Glu residues at position 24. The Δ*G*_sep_^◦^ for the tetramer and dimer steps is more negative at high pH, and could be attributed to disruption of salt bridges, which are not present at low pH due to all Glu residues being protonated. The fact that the Δ*G*_sep_^◦^ values of the pentamer and trimer steps are equal at low and high pH could be attributed to the disruption of hydrogen bonds at low pH and salt bridges at high pH balancing each other.

The  term contributes crucially to the Δ*G*_bind_^◦^ value, affecting the overall sign of Δ*G*_bind_^◦^ of each step. At low pH, it is negative for the hexamer and pentamer states, indicating that the “receptor” helices in each case have more rearrangement freedom in the bound state. For the pentamer intermediate state, the absence of one helix is likely causing the remaining helices to seek to reorganize and reinstate the disrupted hydrogen bond network. Since the “receptor” in this case is composed of four helices in an “open” parallel conformation, it is unlikely that this “receptor” complex would want to rearrange in the bulk to form a more coiled-coil-like tetramer structure, given that the antiparallel tetramer state is accessed at high pH only. With regards to the hexamer state, while at first glance a negative  value seems counterintuitive given the hexamer at low pH is the experimentally observed structure, it should be noted that the E and F helices show C-terminal fraying, with residues missing from the structure;^29^ as a consequence, the hexamer likely has increased conformational plasticity and has the potential to rearrange its helices in the bound state to maximize hydrogen bond formation at the core. This may be less likely at high pH, due to repulsions from the negatively charged Glu residues at the core, and as such the  value for the hexamer step at high pH is positive. At high pH, the  is negative for the tetramer, trimer and dimer steps; the fact that it is negative for the tetramer step is consistent with the fact that the “open” parallel conformation must be unstable and would like to rearrange to form the experimentally observed antiparallel tetramer.

An important observation from all binding free energies computed in this work is the range of values obtained for Δ*G*_bind,total_^◦^/helix. For CC-Di, CC-Tri and CC-Tet, it can be seen that Δ*G*_bind,total_^◦^/helix < −10 kcal mol^–1^ (with the exception of the dimeric CC-Tet at −9.5 ± 0.8 kcal mol^–1^, whose dimer interface closely resembles one of the CC-Tet interfaces, Figures S21 and S22). On the contrary, for the pH-switchable CC-Hex*-L24E system, Δ*G*_bind,total_^◦^/helix ≃ −5.5 kcal mol^–1^ for the favorable states. The difference in these values reflects the inherent stability of the complexes; CC-Di, CC-Tri and CC-Tet are hyper-stable coiled coils, with high melting points,^33^ whereas the CC-Hex*-L24E system has increased plasticity to allow conformational switching upon changes in the pH and consequently has a lower Δ*G*_bind,total_^◦^/helix value. These numbers could therefore serve as a valuable heuristic when testing potential coiled-coil designs. We note that apCC-Di, which experimentally only accesses the antiparallel dimer state, also has a lower Δ*G*_bind,total_^◦^/helix (−5.3 ± 0.5 kcal mol^–1^) compared to the other single-state coiled coils, but it has a lower melting point.^89^

## Conclusion

We have demonstrated a novel protocol for computing the binding free energies of coiled-coil complexes using a combination of orientational, positional, helical, and RMSD restraints. The use of Boresch restraints, instead of the Euler and polar angles employed in the original geometrical route framework,^67^ makes the implementation of our approach straightforward in most MD packages. The use of helical and RMSD restraints significantly accelerates the convergence of the free energy calculations and avoids artifacts commonly observed in implicit solvent modeling of interactions between charged side chains, which are frequently found in coiled coils. The viability of our protocol with implicit solvent force fields is of particular importance for its applicability to protein design pipelines, which require computationally efficient protocols to screen large numbers of designs. We have shown that our approach can successfully recover the preferred topological arrangement of different coiled coils in a given oligomerization state, the preferred oligomerization state of different coiled coils, and pH-dependent conformational effects. Our approach could be used to assess the viability of designs produced by faster, but less accurate, parametric design pipelines, thereby increasing the likelihood of success in multistate protein design efforts.

There are several avenues that could be explored to further develop our approach. First, our method does not account for the folding free energy of isolated monomers into helices prior to their assembly. This could be addressed via enhanced sampling MD protocols to compute folding free energies,^119−121^ or alternatively, predicted through machine learning approaches.^122^ Although we only examined homomultimeric coiled coils, composed of monomers with identical sequences, our approach could also be readily applied to heteromultimeric coiled coils, without the symmetry correction term. Studies of heteromultimeric coiled coils should account for the differences in helical propensities of the individual monomers. The current protocol relies on having plausible starting structures for the coiled-coil assemblies being simulated. This is realistically achievable for coiled coils given the wealth of sequence-to-structure relationships available for this protein fold, with the most plausible topology and oligomerization state for a given sequence obtainable via parametric modeling. If a given sequence is simulated with an unrealistic topology and/or oligomerization state, the computed Δ*G*_bind,total_^◦^/helix value should indicate that the structure is unfavorable, especially when compared to Δ*G*_bind,total_^◦^/helix values of other favorable and unfavorable assemblies. The simulation speeds for the current implicit solvent protocol range from 1350 ns/day for a dimer separation step to 670 ns/day for a hexamer separation step in the presence of all restraints on a NVIDIA RTX3080 GPU. All PMF calculations were run for uniform amounts of time to ensure convergence of the angle and dihedral equilibrium values and the free energy profiles, however it may be enough to run them for shorter time scales, which could significantly reduce simulation costs. The present work used the AMBER ff96 force field in combination with the OBC2 implicit solvent model, which has been successfully applied to study the association of proteins and peptides by others.^123−125^ However, implicit solvent models have well-known limitations that may hinder applicability to all classes of protein–protein interactions. Implicit solvent models struggle to capturing the length-scale dependence of hydrophobic interactions,^126^ will poorly account for the energetics of protein–protein interactions mediated by interfacial water molecules, and potentially protein–protein interfaces with numerous salt bridges; in such scenarios, explicit solvent modeling protocols may be necessary. While our approach can be used in explicit solvent simulations, the increased computational cost to achieve converged binding free energies may limit its scalability for large-scale applications. An interesting direction would be to derive transferable implicit solvent models using machine learning, trained on explicit solvent trajectories.^127,128^ We have also limited our pH studies to low and high pH conditions with fixed protonation states. A more realistic treatment of pH effects could be achieved by coupling our approach with constant-pH MD methods^129−131^ to describe coiled-coil assemblies at pH values where a mixture of distinct protonation states is expected to coexist. Finally, our methodology could be applied to other classes of protein–protein interactions by modifying the protocols used to apply helical or RMSD restraints. We hope that our modified geometrical route will enable the study of systems of interest for biotechnological and pharmaceutical applications.

## Data Availability

Parametric design code and designed coiled-coil structures, Boresch restraints selection and analysis code, restraint anchor points, example input files to run Boresch-restrained metadynamics and free energy analysis code are available on GitHub at michellab/CCmetaD.

## Supporting Information Available

Derivation of the symmetry correction for multimeric complexes, coiled-coil monomer simulations and end-to-end distance calculations with different force fields and implicit solvent models, unrestrained metadynamics PMFs, funnel metadynamics implementation, PMFs and interhelical contact maps from funnel metadynamics, Boresch-restrained metadynamics free energy values, example PMFs and interhelical contact maps (PDF)
