# Supplementary material for: Assessment of the Topology and Oligomerisation States of Coiled Coils Using Metadynamics with Conformational Restraints
Source: J Chem Theory Comput. 2025 Mar 5;21(6):3260–76. doi: 10.1021/acs.jctc.4c01695 (PMC11948332; doi:10.1021/acs.jctc.4c01695)
Supplement: Supplementary file 1 — ct4c01695_si_001.pdf [file ct4c01695_si_001.pdf]

Supplementary Information: Assessment of the Topology and Oligomerisation States of Coiled Coils Using Metadynamics with Conformational Restraints

Evangelia Notari,[†] Christopher W. Wood,[‡] and Julien Michel[*,†]

†*EaStCHEM School of Chemistry, University of Edinburgh, David Brewster Road, Edinburgh EH9 3FJ, United Kingdom*

‡*School of Biological Sciences, University of Edinburgh, Roger Land Building, Edinburgh EH9 3FF, United Kingdom*

E-mail: julien.michel@ed.ac.uk

1. Symmetry Correction

The effect of symmetry on the calculation of binding free energies must be taken into account when the bound complex is composed of identical monomers, as is the case in homomeric coiled coils. Duboué-Dijon et al. devised a thought experiment to showcase the differences between the binding constants of homo- and heterodimerisation.[1] Here we generalise this thought experiment to homo- and heteromultimerisation, starting from the homo- and heterodimerisation as described by Duboué-Dijon et al.[1]

Consider a dilute solution containing molecules that can either be identical (M) or distinct (A, B in the case of dimerisation, A, B, C in the case of trimerisation etc., where it is assumed that the distinct monomers have the same behaviour during the multimerisation process). We

also assume that the system obeys the law of mass action,[2,3] and that the total concentration of each monomer A, B, C etc. is equal to 1 M.

We first examine the heterodimerisation equilibrium:

$$A + B \leftrightarrow AB$$

The possible dimers that can be formed from the A and B monomers are $2^2 = 4$ (AB, BA, AA and BB), out of which $2! = 2$ correspond to the heterodimer (AB and BA). Therefore, the heterodimer has twice the concentration of each homodimer. If we define [AA] = [BB] = x, then [AB] = [BA] = 2x, and [A] = [B] = 1-4x. From the law of mass action for the heterodimerisation:

$$K^{\circ}_{\mathrm{AB}} = \frac{[AB]C^{\circ}}{[A][B]} = \frac{2xC^{\circ}}{(1-4x)^2} \quad (1)$$

In the case of homodimerisation, where A and B have identical labels M, the equilibrium is:

$$2M \leftrightarrow M_2$$

Here, $[M_2]$ = [AB] + [AA] + [BB] = 4x, and [M] = [A] + [B] = 2(1-4x). From the law of mass action for the homodimerisation:

$$K^{\circ}_{\mathrm{M_2}} = \frac{[M_2]C^{\circ}}{[M]^2} = \frac{4xC^{\circ}}{[2(1-4x)]^2} \quad (2)$$

From eq. 1 and 2:

$$K^{\circ}_{\mathrm{M_2}} = \frac{1}{2}K^{\circ}_{\mathrm{AB}} \quad (3)$$

We then examine the hetero- and homotrimerisation cases. In heterotrimerisation:

$$A + B + C \leftrightarrow ABC$$

The possible trimers that can be formed from the A, B, C monomers are $3^3 = 27$, out of which $3! = 6$ correspond to the heterotrimer (ABC, ACB, BAC, BCA, CAB, CBA). Therefore, the heterotrimer has six times the concentration of all the other possible trimers (e.g. AAA, AAB, AAC etc.). If we define [AAA] = [BBB] = [CCC] = x, then [ABC] = 6x, and and [A] = [B] = [C] = 1-27x. From the law of mass action for the heterotrimerisation:

$$K^{\circ}_{\mathrm{ABC}} = \frac{[ABC]C^{\circ}}{[A][B][C]} = \frac{6xC^{\circ}}{(1-27x)^3} \tag{4}$$

In the case of homotrimerisation, where A, B and C have identical labels M, the equilibrium is:

$$3M \leftrightarrow M_3$$

Here, $[M_3]$ = 27x, and [M] = [A] + [B] + [C] = 3(1-27x). From the law of mass action for the homotrimerisation:

$$K^{\circ}_{\mathrm{M_3}} = \frac{[M_3]C^{\circ}}{[M]^3} = \frac{27xC^{\circ}}{[3(1-27x)]^3} \tag{5}$$

From eq. 4 and 5:

$$K^{\circ}_{\mathrm{M_3}} = \frac{1}{6}K^{\circ}_{\mathrm{ABC}} \tag{6}$$

It can be generalised from the above that in the case of heteromultimerisation of n distinct molecules into a complex X, the equilibrium constant will have the general form:

$$K^{\circ}_{\mathrm{X}} = \frac{n!xC^{\circ}}{(1-n^n x)^n} \tag{7}$$

wheras in the case of homomultimerisation of n identical molecules into a complex M_n, the equilibrium constant will have the general form:

$$K^{\circ}_{\mathrm{M_n}} = \frac{n^n x C^{\circ}}{[n(1-n^n x)]^n} \tag{8}$$

From eq. 7 and 8:

$$K^{\circ}_{\mathrm{M_n}} = \frac{1}{n!} K^{\circ}_{\mathrm{X}} \tag{9}$$

As a consequence, when calculating the binding free energy of complexes composed of n identical monomers, an additional term equal to $k_B T ln(\frac{1}{n!})$ ought to be taken into account.

2. Design of Coiled Coils with Alternative Topologies and Oligomerisation States

The following coiled coils were parametrically designed in ISAMBARD:[4] CC-Di with parallel and antiparallel topologies, apCC-Di with parallel topology, CC-Tri with up-down-up topology, CC-Di as a parallel trimer and tetramer, CC-Tri as a parallel dimer and tetramer, and CC-Tet as a parallel dimer and trimer.

All four available design algorithms (genetic algorithm, differential evolution, particle swarm optimisation and covariance matrix adaptive evolutionary strategy) were employed for each design. Designs for simulations were chosen based on their BUDE scores and on the convergence of the design process. This was assessed from funnel plots of all generated designs, where each design is compared to the best design (the one with the lowest BUDE score) by means of their RMSDs (see Figure 1 in the main text.)

CC-Di was designed in its native parallel dimer conformation to compare with simulations of the X-ray structure. Aligning the two structures in PyMOL[5] results in an RMSD of 0.76 Å (Figure S1). The biggest difference between the two structures is the rotamer ori-

entations of residues pointing towards the solvent, which will nevertheless fluctuate during the simulations.

The designed structures and their respective coiled coil parameters can be found on GitHub at michellab/CCmetaD.

Table S1: Sequences of the *de novo* designed coiled coils studied and coiled coil register of each sequence.

**Peptide**	**Sequence**	**Register**
CC-Di	Ac-GEIAALKQEIAALKKENAALKWEIAALKQGYY-NH_2	*fgabcde*
apCC-Di	Ac-GQLEQELAALDQQIAALKQRRAALKWQIQG-NH_2	*bcdefga*
CC-Tri	Ac-GEIAAIKQEIAAIKKEIAAIKWEIAAIKQGYG-NH_2	*fgabcde*
CC-Tet	Ac-GELAAIKQELAAIKKELAAIKWELAAIKQGAG-NH_2	*fgabcde*
CC-Hex*-L24E	Ac-GELKAIAQELKAIAKELKAIAWEEKAIAQG-NH_2	*fgabcde*

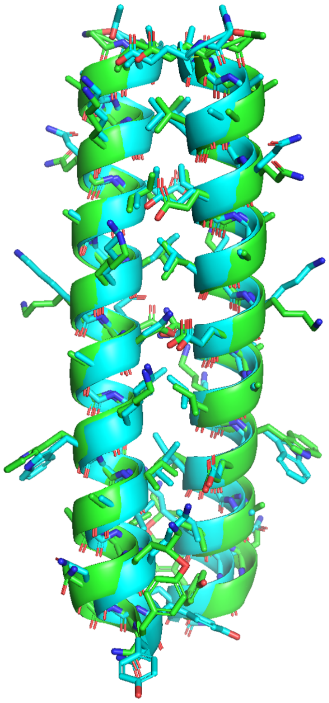

Figure S1: CC-Di X-ray structure (green) aligned with designed ISAMBARD structure (cyan), with an RMSD of 0.76 Å. Rendered with PyMOL.[5]

3. Coiled Coil Monomer Simulations with Different Force Fields and Implicit Solvent Models

Since the coiled coil oligomerisation process was computationally decoupled in folding and association, we were interested in preserving the helicity of coiled-coil monomers when unbound from the complex. The sequences of coiled-coil monomers generally have low helical propensities, and therefore the monomers are not fully folded, but rather form partially helical regions. Interactions between these partially formed α-helices are the driving force for oligomerisation.[6] As a consequence, any dissociated monomer is expected to unfold. This behaviour was verified with short MD simulations of monomers from each of the coiled coils studied (Table S1), carried out with the ff14SBonlysc force field and the GBn2 model, which is the recommended combination for implicit solvent simulations.[7–9] From these simulations, it was observed that the α-helices would collapse and form globular-like structures, with reduced helicity and linearity (Figure S2). We therefore sought to identify a force field and implicit solvent combination that would best retain the desired α-helical structure of the monomers when separated from the assembly.

The force fields studied were ff94,[10] ff96,[11] ff98,[12] ff99SB,[13] ff99SBnmr,[14] ff14SB[15] and ff14SBonlysc[15] and the implicit solvent models studied were OBC2,[16] GBn[17] and GBn2.[7] All 21 possible combinations were evaluated on their ability to retain the helicity and linearity of the monomer helices, with the end-to-end distance between the first and last Cα of each α-helix as the evaluation metric. A single α-helix was extracted from each of the coiled coils studied (Table S1), and parameterised for each force field and solvent combination with the tleap module of AmberTools 22.[18] Three independent MD simulations of 200 ns were run for each monomer and combination in OpenMM 7.5.[19] The simulations were run at 298.15 K employing a Langevin thermostat with a damping coefficient of 1 ps^{-1}. Hydrogen mass repartitioning was used, with the mass of each hydrogen atom set to 1.5 amu. The integration timestep was 4 fs. The end-to-end Cα distance was computed with MDTraj.[20] In order to

allow comparisons between monomers with different numbers of residues, the Cα distance was divided by the ideal length of an α-helix of a given number of residues, taking into account that the rise per residue in α-helices is 1.5 Å.[21] From this analysis (Figure S3), it became apparent that the ff96/OBC2 combination was the most adequate choice to retain monomer helicity and linearity. This is consistent with previous studies reporting that this combination tends to stabilise α-helical structures.[22–24] However, it should be noted that ff96/OBC2 only manages to achieve 51% of the ideal helix length, and generally all combinations tested result in partially unfolded structures to different extents; this is not unexpected given the low helical propensity of coiled-coil monomers. The ff96/OBC2 combination was used for all subsequent metadynamics simulations and free energy calculations.

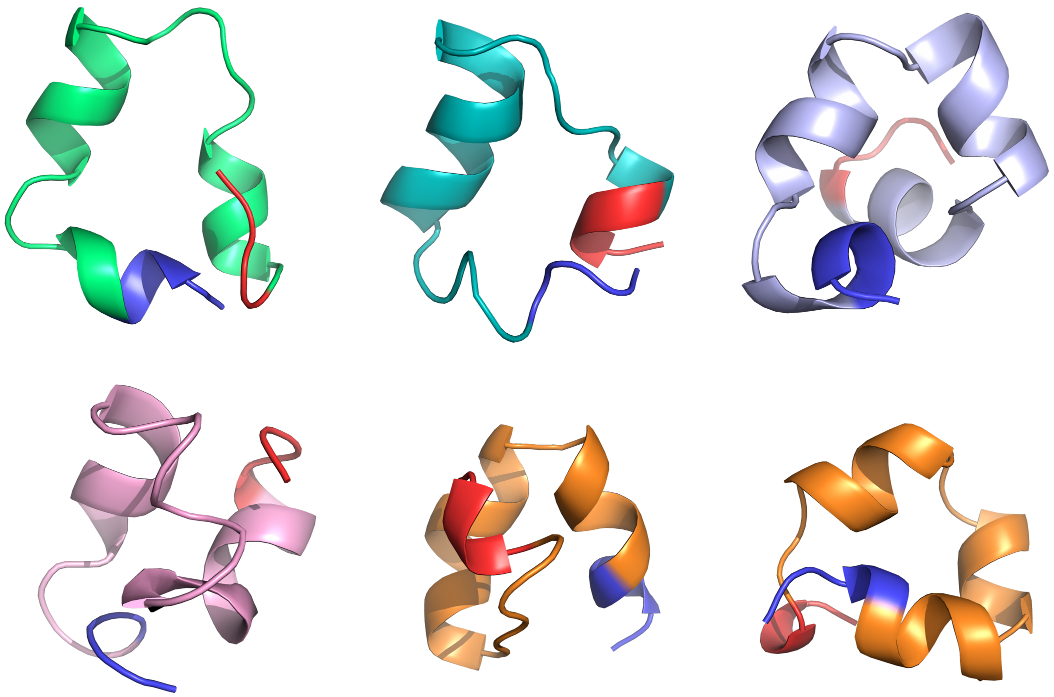

Figure S2: Coiled-coil monomer structures from simulations run with the ff14SBonlysc force field and the GBn2 implicit solvent model. Representative structures were clustered from the simulations using Ward hierarchical clustering,[25] as implemented in MDTraj.[20] Green: CC-Di, cyan: apCC-Di, purple: CC-Tri, pink: CC-Tet, orange: CC-Hex*-L24E hexamer (left) and tetramer (right). N-termini are coloured in blue and C-termini are coloured in red. Rendered with PyMOL.[5]

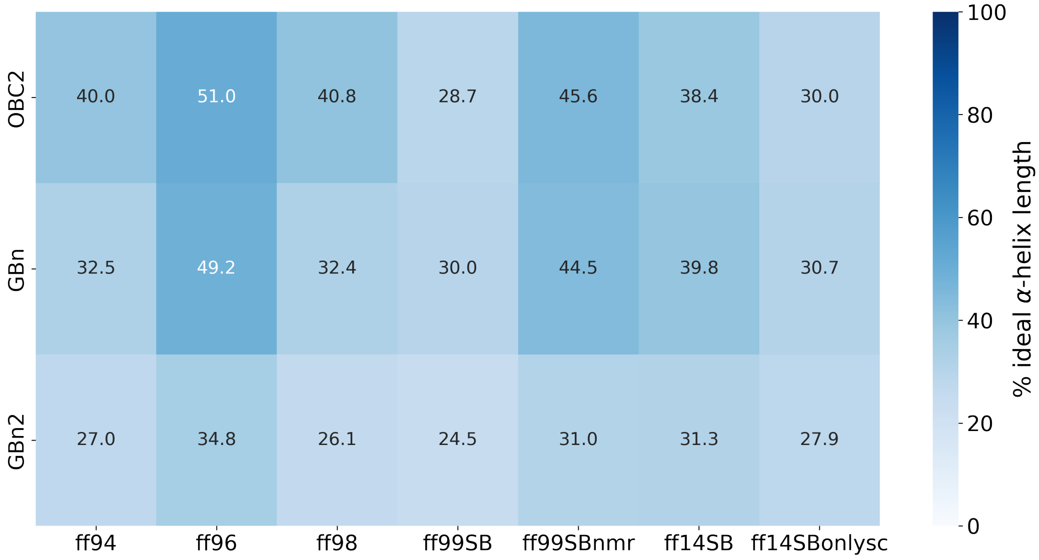

Figure S3: End-to-end distance of first and last Cα atoms divided by the ideal α-helix length for the number of residues in each sequence.

4. Metadynamics Simulation Protocols

Helical ϕ and ψ Torsion Restraints

Initial test metadynamics simulations of coiled-coil dimers with ff96/OBC2 and the distance r between the centres of mass of the monomers as the CV showed that even when using the most promising force field and solvent combination, the dissociated monomers still unfold partially. It was therefore decided to impose restraints on the ϕ, ψ torsions of each α-helix, in order to prevent unfolding in the unbound state. Flat bottom restraints were imposed on each quartet of atoms forming a ϕ or ψ torsion, with a force constant of 100 kJ mol^{-1}. The ϕ, ψ torsions were restricted in the right-handed α-helical region of the Ramachandran plot. It should be noted that these helical restraints are not accounted for during the calculation of the association free energy, with the cost of folding each α-helix taken into consideration implicitly by normalising the association free energy with the number of helices in the assembly. While in principle any force field and implicit solvent model can be used along with these helical restraints, using ff96/OBC2 which favours α-helical structures is expected to lead to the least structural distortion of the fully folded helical monomers in the unbound state. All subsequent metadynamics simulations were run in the presence of these helical ϕ, ψ restraints. Ramos et al.[26] also employed harmonic restraints on the backbone Cα atoms of coiled coils to maintain the desired helical fold, and then divided their energy calculations by the number of helices in the assembly.

Sequential Removal of α-Helices

For oligomerisation steps higher than dimers, the association step was decomposed into sequential, distinct steps (see Figure 1 in the main text), where one α-helix at a time is removed from the complex. The first chain of each PDB entry is conventionally labelled as the 'ligand' and the remaining chains are labelled as the 'receptor', with the definitions updated accordingly for each intermediate state. For example, in the coiled-coil trimer CC-

Tri, chain A is the 'ligand' and chains B-C are the 'receptor' in the trimer separation step. In the subsequent B-C dimer separation step, chain B is labelled as the 'ligand' and chain C is labelled as the 'receptor'. The only exception to this labelling convention was the designed up-down-up variant of CC-Tri, which was separated via two different schemes to confirm that the order by which the monomers are separated does not affect the free energy calculations (Figure S4). In scheme 1, the conventional labelling is retained, with chain A ('up') being separated from the 'up-down' BC complex first, followed by chain B ('down') separated from chain C ('up'). In scheme 2, chain B ('down') is separated from the 'up-up' AC complex, followed by chain A separated from chain C.

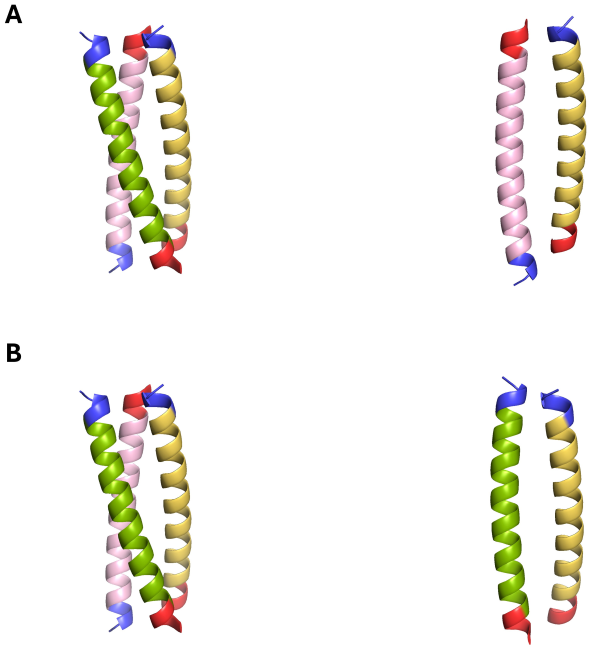

Figure S4: The two separation schemes for the up-down-up variant of CC-Tri. Helix A is shown in green, helix B is shown in pink and helix C is shown in yellow. The N-termini and C-termini are coloured blue and red respectively. **A**. Scheme 1: In the trimer dissociation step (left), helix A is separated from helices B and C. In the dimer dissociation step (right), helix B is separated from helix C. **B**. Scheme 2: In the trimer dissociation step (left), helix B is separated from helices A and C. In the dimer dissociation step (right), helix A is separated from helix C. Rendered with PyMOL.[5]

Unrestrained Metadynamics Simulations Setup and PMF Profiles

All metadynamics simulations were initiated from pre-equilibrated structures. The metadynamics parameters chosen were an initial Gaussian height of 0.8 kJ mol^{-1}, a Gaussian width of 0.025 nm and a bias factor of 20. Biases were deposited every 0.4 ps. The CV was the distance r between the centres of mass of the two monomers. Representative structures were recovered from the simulation trajectories with hierarchical clustering[25] as implemented in MDTraj.[20] Inter-helical interactions were computed with the getcontacts library.[27]

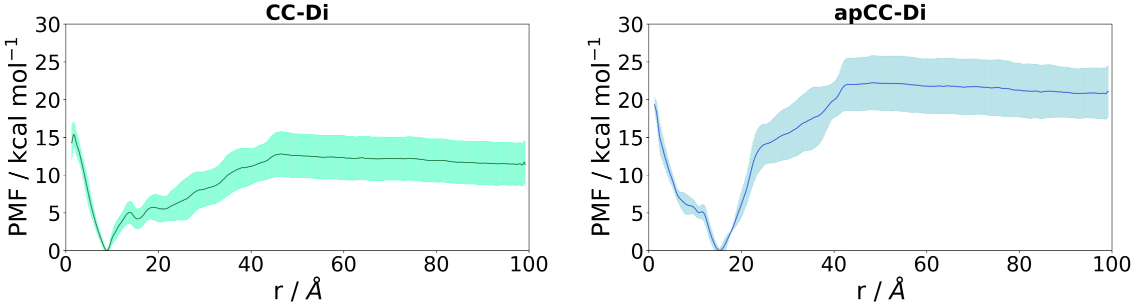

Figure S5: PMF profiles from unrestrained metadynamics simulations of CC-Di and apCC-Di. The shaded areas represent standard error from 3 independent replicates of 1 μs each. The only restraints present are the helical ϕ, ψ restraints. The absence of positional and orientational restraints results in limited reproducibility among independent replicates.

Funnel Metadynamics Setup and Analysis

The funnel metadynamics implementation was inspired by the approach followed in Hedges et al.[28] and was further customised for OpenMM.[19] The funnel is constructed with three reference points: the centre of mass of helix A, the centre of mass of helix B and the last Cα atom of helix B. The funnel shape is defined by the following equation:

$$S = h\left(\frac{1}{1+e^{b(y-a)}}\right) + f \qquad (10)$$

where S is the funnel radius at any value of y, h is the wall width, a is the location of the funnel inflection point, b is the steepness at the inflection point and f is the wall buffer

(Figure S6). In the case of dimeric coiled coils, the CV was the distance r between the centres of mass of the two monomers. For the higher oligomerisation states, the CV in each separation step is the distance r between the centres of mass of the 'ligand' and 'receptor' helices. The metadynamics parameters chosen were an initial Gaussian height of 0.8 kJ mol^{-1}, a Gaussian width of 0.025 nm and a bias factor of 20. Biases were deposited every 0.4 ps. Funnel restraints restrict the space available to the coiled-coil monomers along the x axis, and upper and lower wall restraints restrict the space along the y axis.

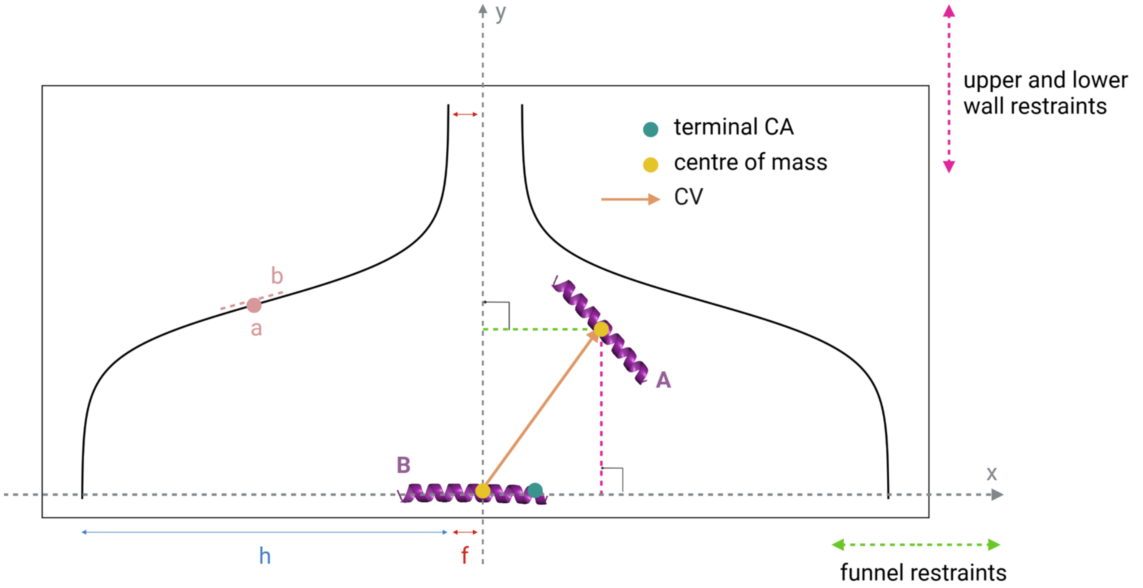

Figure S6: Funnel design and parameters for the funnel metadynamics simulations. Refer to the text for a description of each variable. Note that the funnels used in the simulations were smaller, with the wall width h being equal to or smaller than the length of a monomer helix. A larger funnel is depicted here for clarity.

Funnel metadynamics simulations were run for CC-Di, apCC-Di and CC-Tri to examine whether the limited reproducibility observed in the unrestrained metadynamics simulations could be mitigated by restraining the space available to the monomers when dissociated. Different values for h, f, a and b were tested for each coiled coil. The wall width h values examined were equal to the length of a coiled coil monomer, or fractions of it (1/2, 1/3 or 1/4 of monomer length). The wall buffer f values examined were 0.5 nm or 1 nm. The inflection point a and inflection steepness b values were kept at 5 nm and 1.5, respectively.

Different force constants for the funnel restraints were examined in the range 1-100 kJ mol^{-1} nm^{-2}. The force constant for the upper and lower wall restraints was set to 1000 kJ mol^{-1} nm^{-2}. Results from selected funnel setups are shown in Figure S7. The reproducibility between independent replicates appears to be affected by the funnel parameters and/or fortuitous sampling. Representative structures were recovered from the simulation trajectories with hierarchical clustering[25] as implemented in MDTraj.[20] Inter-helical interactions were computed with the getcontacts library.[27]

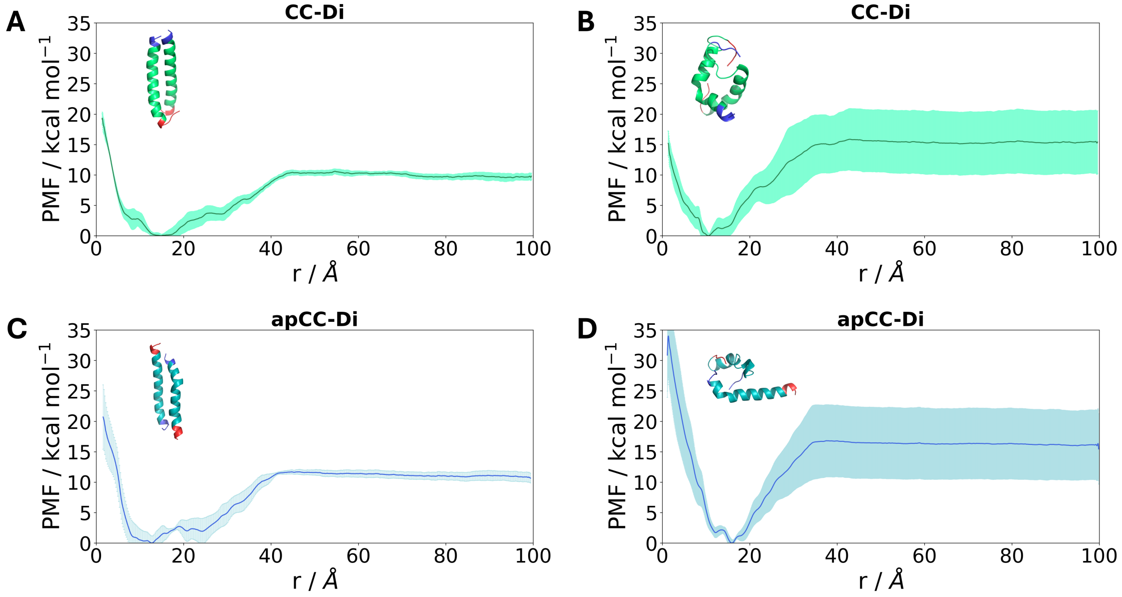

Figure S7: PMF profiles from funnel metadynamics simulations of CC-Di and apCC-Di. The shaded areas represent standard error from 3 independent replicates of 1 μs each. Representative structures from clustering the bound conformations of the dimers are also shown. **A**. CC-Di with a funnel wall width of $0.5 \cdot$ coiled coil length and a wall buffer of 0.5 nm. **B**. CC-Di with a funnel wall width of $0.33 \cdot$ coiled coil length and a wall buffer of 1 nm. **C**. apCC-Di with a funnel wall width of $0.25 \cdot$ coiled coil length and a wall buffer of 1 nm. **D**. apCC-Di with a funnel wall width of $0.5 \cdot$ coiled coil length and a wall buffer of 0.5 nm. The force constant for the funnel restraints was 1 kJ mol^{-1} nm^{-2}.

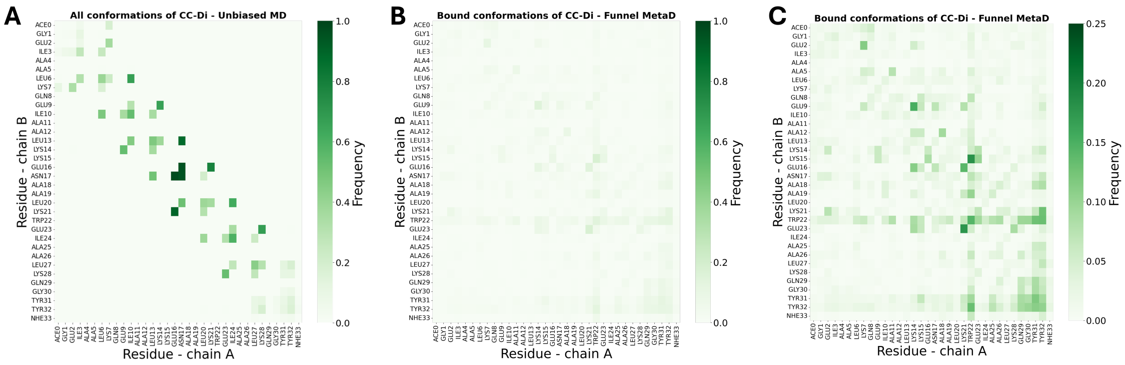

Figure S8: Intermolecular interactions between chains A and B in CC-Di. **A**. Intermolecular interactions from unbiased MD simulations of the bound complex. **B**. Intermolecular interactions of the bound conformations of CC-Di from funnel metadynamics simulations. **C**. Intermolecular interactions of the bound conformations of CC-Di from funnel metadynamics simulations, where the interaction frequency has been capped at 25%, as no unique interaction was present more than 25% of the simulation frames where CC-Di is in the bound state.

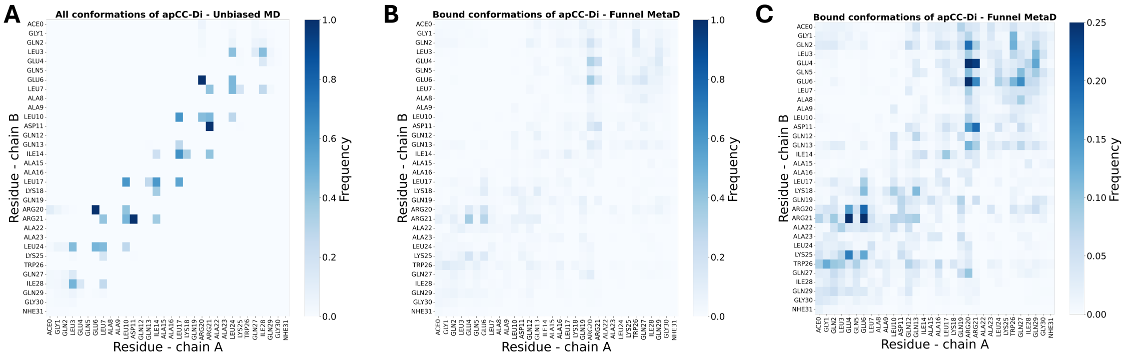

Figure S9: Intermolecular interactions between chains A and B in apCC-Di. **A**. Intermolecular interactions from unbiased MD simulations of the bound complex. **B**. Intermolecular interactions of the bound conformations of apCC-Di from funnel metadynamics simulations. **C**. Intermolecular interactions of the bound conformations of apCC-Di from funnel metadynamics simulations, where the interaction frequency has been capped at 25%, as no unique interaction was present more than 25% of the simulation frames where apCC-Di is in the bound state.

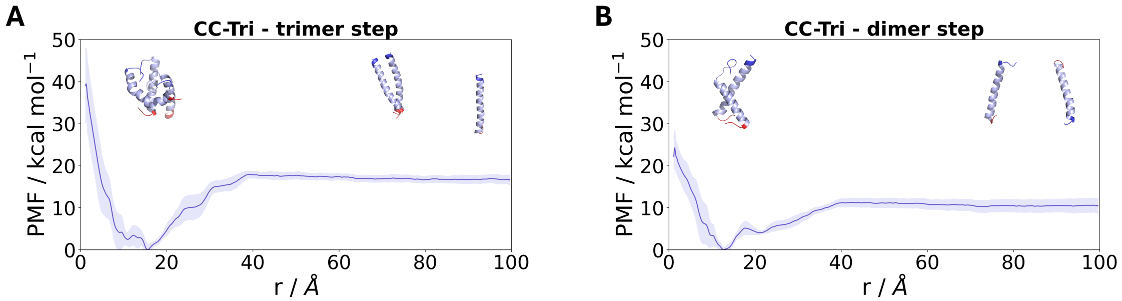

Figure S10: PMF profiles from funnel metadynamics simulations of CC-Tri, carried out in two steps. The shaded areas represent standard error. Representative structures from clustering the bound and unbound conformations of the assemblies are also shown. Both steps were carried out with a funnel wall width of 0.25 · coiled coil length and a wall buffer of 1 nm. **A**. The separation step of CC-Tri into a dimer (chains B-C) and a monomer (chain A). **B**. The separation of the remaining dimer (chains B-C).

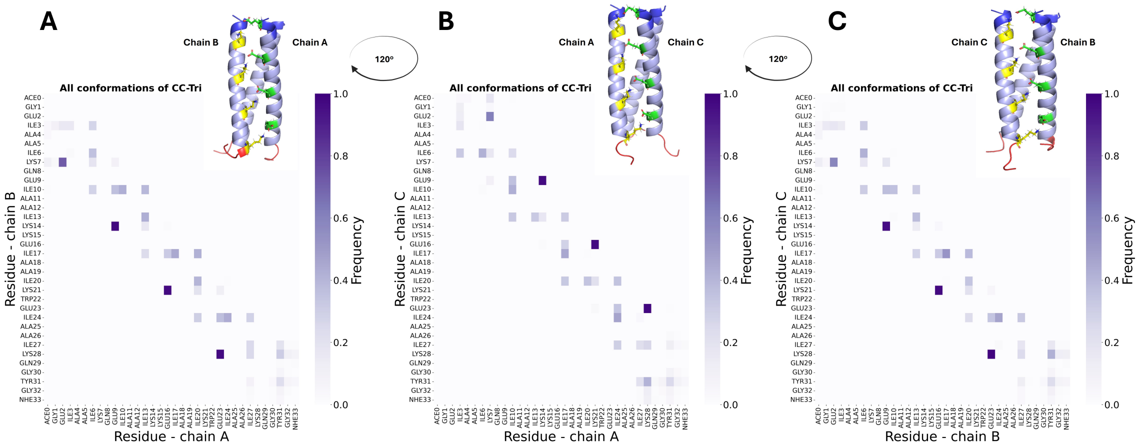

Figure S11: Intermolecular interactions between pairs of chains from unbiased simulations of the bound complex of CC-Tri. Each inset shows the respective coiled coil interface depicted in each heatmap, with residues forming salt bridges depicted with green (Glu) and yellow (Lys). **A**. Interactions between chains A and B. **B**. Interactions between chains A and C. **C**. Interactions between chains B and C.

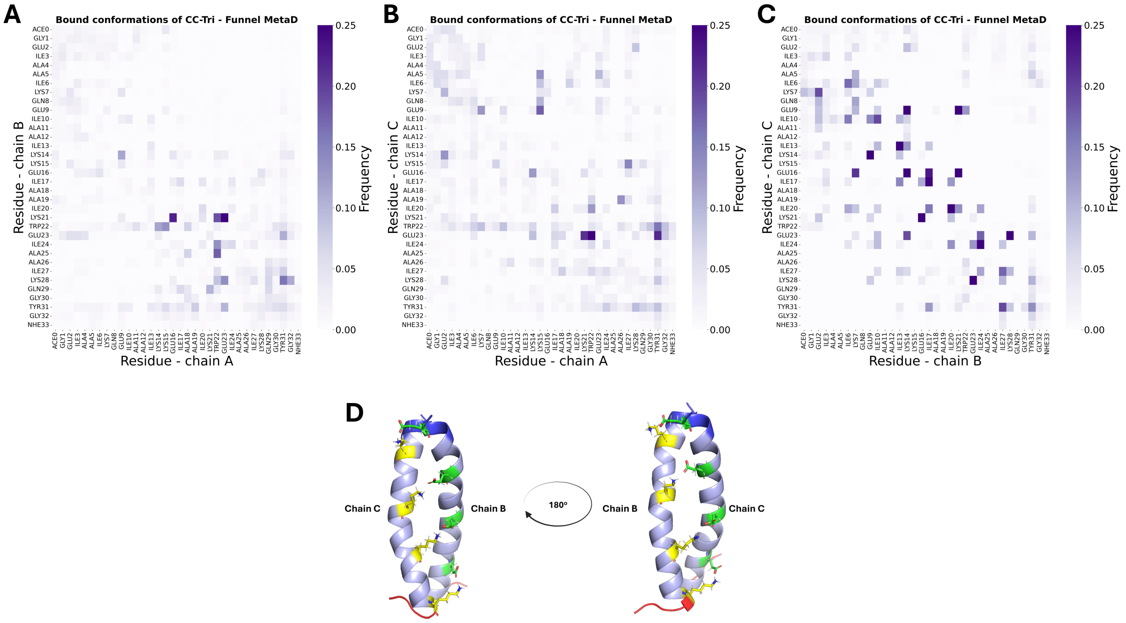

Figure S12: Intermolecular interactions between pairs of chains from funnel metadynamics simulations of the bound conformations of CC-Tri. The interaction frequency has been capped at 25%, as no unique interaction was present more than 25% of the simulation frames where CC-Tri is in the bound state. **A**. Interactions between chains A and B. **B**. Interactions between chains A and C. **C**. Interactions between chains B and C. **D**. Re-arrangement of the 'receptor' chains B and C in the absence of chain A. This results in the formation of salt bridges between Glu (green) and Lys (yellow) residues on both sides of the complex, which is characteristic of coiled-coil dimers.

5. Metadynamics with Boresch Restraints

Selection of Restraint Anchor Points

In order to select anchor points for the Boresch restraints, implicit solvent simulations of the complexes were run for 200 ns with three independent replicates, with the same starting conformation but different starting velocities. In the case of intermediate states from assemblies larger than dimers, where one or more α-helices have been removed from the complex, an additional RMSD restraint was applied on the backbone atoms of the intermediate complex to prevent re-organisation of the helices and to facilitate the selection of restraints that would preserve the desired 'open' conformation. If the helices are allowed to rearrange in the absence of a missing chain, the resulting complex can adopt a 'closed' conformation (Figure S13). Selecting restraints for this 'closed' conformation will result in the stabilisation of a different intermediate state, which will result in the calculation of an erroneous binding free energy.

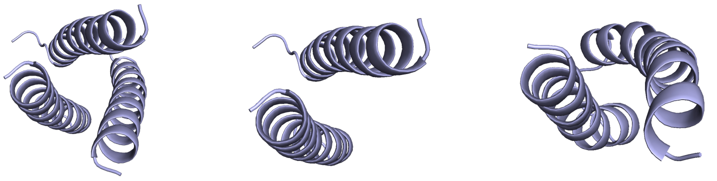

Figure S13: Left: CC-Tri X-ray structure, viewed from the N- to the C-terminus. Middle: 'Open' conformation of the CC-Tri 'receptor' helices B-C. Right: 'Closed' conformation of the CC-Tri 'receptor' helices B-C, forming a structure that resembles a coiled-coil dimer.

The trajectories were then analysed with MDRestraintsGenerator[29,30] to identify the most stable regions in the structure; selecting anchor points in highly flexible regions of the complex should be avoided, as this can result in systematic errors and/or numerical instabilities and thus negatively affect the convergence and accuracy of the free energy calculations.[31–34] Three Cα atoms were then picked on the 'ligand' and three on the 'receptor' to construct the frame of reference for the restraints. The distributions of the Boresch angles and torsions were examined in each case, and care was taken to avoid three contiguous

atoms being close to collinearity (0° or 180°), as this can potentially cause numerical instabilities and simulation crashes due to large torsional forces, a known limitation of Boresch restraints.[34,35] We limited the selection of anchor points to Cα atoms, as this reduces the restraint search space and allows for the exploitation of coiled coil symmetry. Selecting Cα atoms from alternating faces of the α-helix in stable regions of the assembly is a simple heuristic for identifying Boresch restraints that avoid collinearity. The anchor points chosen for each coiled coil and intermediate state, along with equilibrium values, can be found on GitHub at michellab/CCmetaD. An example of selected anchor points, distributions of the Boresch angles and torsions and PMF profiles from each CV is shown in Figure 3 in the main text for the apCC-Di dimer.

Geometrical Route with Boresch Restraints

The geometrical route with the Boresch restraints was run as a series of sequential metadynamics calculations on each CV to obtain its PMF. All metadynamics simulations were initiated from pre-equilibrated structures. There are two variations of the protocol, depending on the coiled coil assembly:

For dimeric coiled coils, the CVs are the Boresch angles and dihedrals θ_A, θ_B, ϕ_A, ϕ_B, ϕ_C and the distance between the centres of mass r of the 'ligand' and 'receptor' helices. The initial PMF calculation on θ_A is carried out in the absence of any restraints (except the helical ϕ, ψ restraints). The subsequent PMF calculation on θ_B is carried out in the presence of the θ_A restraint, with the θ_A equilibrium value updated to the value at the minimum of its PMF. The PMF calculation on ϕ_A is carried out in the presence of the θ_A and θ_B restraints (with the θ_B equilibrium value also updated), and so on. This leads up to the separation PMF, where all Boresch angles and dihedrals have been restrained to their equilibrium values. We note that this scheme only applies to 'true' coiled-coil dimers, and not to dimeric intermediate states from larger assemblies, which need to be restrained to their 'open' conformations.

For higher oligomerisation states, an additional RMSD restraint is imposed on the backbone atoms of the 'receptor' helices, to prevent their re-organisation in the absence of the dissociating helix. In the absence of this restraint, the 'receptor' helices will re-organise from an 'open' conformation to a 'closed' conformation, which will prevent the dissociated helix from binding correctly upon re-association (Figure S13); this will invalidate the constructed free energy pathway. The series of PMF calculations is initiated with a PMF calculation on the RMSD of the receptor while the complex is in the bound state. The subsequent PMF calculation on θ_A is carried out in the presence of the RMSD restraint (with its equilibrium value set to 0), and so on. The separation PMF is carried out in the presence of the Boresch restraints and the RMSD restraint on the 'receptor'. A final PMF calculation is done on the RMSD of the receptor in the unbound state. This calculation series is repeated for each intermediate state until all helices have been dissociated. Interestingly, we observed that CC-Tri was the only higher order coiled coil in our study which was able to re-organise its two 'receptor' helices from the 'closed' to the 'open' conformation in the simulation timescales to allow the dissociated 'ligand' helix to bind correctly. We therefore ran its binding free energy calculations with and without RMSD restraints. This served as an additional test for our protocol, as the binding free energy obtained with RMSD restraints should be the same as the binding free energy obtained without any RMSD restraints after correcting for the presence of restraints.

The binding free energy for a dimeric coiled coil can be calculated by separating the two α-helices, and using eq. (16) from the main text, including the necessary symmetry correction. Since no conformational (RMSD) restraints are used in the dimer calculations, the $\Delta G_\mathrm{c}^\mathrm{bulk}$ and $\Delta G_\mathrm{c}^\mathrm{site}$ terms are omitted. For higher oligomerisation states, intermediate binding free energies can be obtained from eq. (6) from the main text for each step, omitting the symmetry term. The total binding free energy of the coiled coil is then obtained by adding the binding free energies from each intermediate step, and including the symmetry correction for the full complex at the end.

The metadynamics parameters chosen for the simulations with the Boresch and RMSD restraints were an initial Gaussian height of 0.8 kJ mol^{-1}, a Gaussian width of 0.025 nm for the RMSD and r PMF calculations, a Gaussian width of 0.1 rad for the Boresch angle and dihedral PMF calculations, and a bias factor of 20. Biases were deposited every 0.4 ps. For each PMF calculation, three independent replicates were run for the RMSD, angle and torsion steps, and five replicates were run for the separation steps. Independent replicates had the same starting conformations but different starting velocities. The RMSD, angle and dihedral PMF calculations were run for 400 ns per replicate, and the separation PMF calculations were run for 500 ns per replicate. The force constant for the RMSD restraints was set to 100 kcal mol^{-1} Å^{-2}. The force constants for the Boresch restraints were uniformly set to 200 kcal mol^{-1} rad^{-2}, with the exception of CC-Di (X-ray structure and ISAMBARD-designed structure), apCC-Di, CC-Tri (without RMSD restraints) and the dimer intermediate state of CC-Tet, where the Boresch force constants were set to 100 kcal mol^{-1} rad^{-2}, otherwise numerical instabilities were observed. The convergence of the PMFs was evaluated from monitoring the diffusivity of each CV over the simulation. Occasionally some replicates were observed to be 'stuck' in metastable states for prolonged periods of time, and even sampling of the CV was not achieved over the timescale of the simulation. These problematic runs were easily diagnosed from visualisation of CV diffusivity plots and were discarded (Figure S14). The free energy values from each CV step were calculated with code adapted from the Binding Free Energy Estimator 2 (BFEE2) software,[36,37] which is available at michellab/CCmetaD.

Representative structures were recovered from the simulation trajectories with hierarchical clustering[25] as implemented in MDTraj.[20] Inter-helical interactions were computed with the getcontacts library.[27]

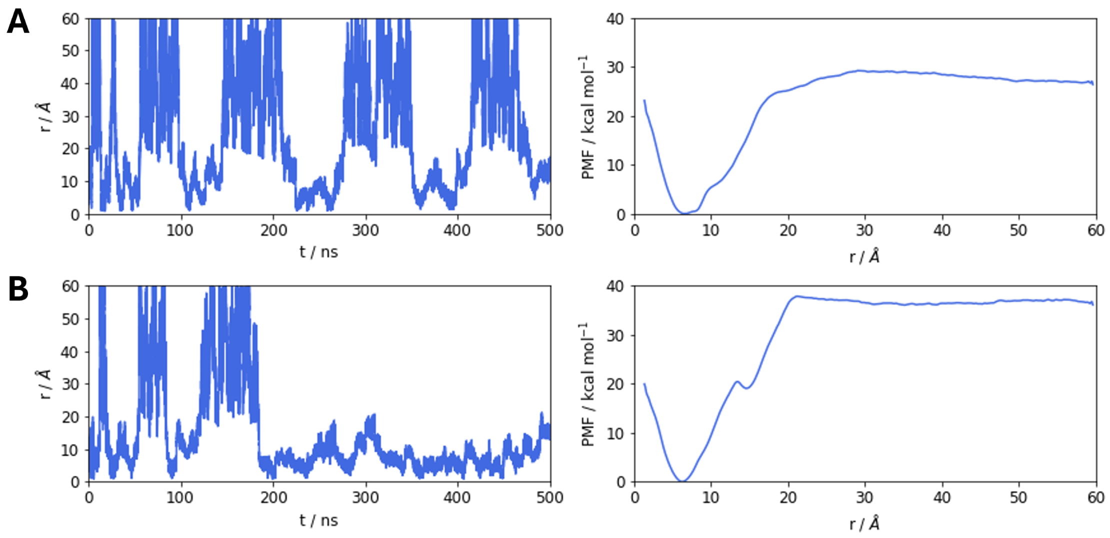

Figure S14: Example Collective Variable and Potential of Mean Force data from separation simulations of CC-Di with conformational, orientational and positional restraints. **A**. A replicate where the bound and unbound states of CC-Di were sufficiently visited over the course of the simulation. This replicate was retained for calculating the $\Delta G^{\circ}_{\text{bind}}$ of the complex. **B**. A replicate where the simulation of CC-Di gets 'stuck' in the bound state for $\simeq$ 300 ns, resulting in an overestimated PMF. This replicate was not used for calculating the $\Delta G^{\circ}_{\text{bind}}$ of the complex.

6. Results from Metadynamics with Boresch Restraints

Table S2: Free energy contributions for the coiled-coil dimers from the modified geometrical route, including the Boresch and symmetry correction terms. *(p)* denotes parallel and *(ap)* denotes antiparallel topologies. Values in kcal mol^{-1}.

**Contribution**	**CC-Di (p)**	**CC-Di (ap)**	**apCC-Di (p)**	**apCC-Di (ap)**
ΔG_{θ_A}	-0.22 ± 0.01	-0.29 ± 0.01	-0.32 ± 0.02	-0.21 ± 0.01
ΔG_{θ_B}	-0.23 ± 0.01	-0.34 ± 0.02	-0.28 ± 0.05	-0.20 ± 0.01
ΔG_{ϕ_A}	-0.30 ± 0.02	-0.37 ± 0.02	-0.52 ± 0.02	-0.27 ± 0.01
ΔG_{ϕ_B}	-0.54 ± 0.04	-0.57 ± 0.01	-0.48 ± 0.01	-0.31 ± 0.01
ΔG_{ϕ_C}	-0.28 ± 0.01	-0.76 ± 0.02	-0.47 ± 0.01	-0.35 ± 0.01
$\Delta G^{\circ}_{\text{sep}}$	-27.6 ± 0.9	-9.7 ± 0.4	-3.4 ± 0.8	-16.5 ± 1.1
$\Delta G^{\circ}_{\text{corr}}$	+6.8 ± 0.1	+8.2 ± 0.1	+7.8 ± 0.1	+6.8 ± 0.1
ΔG_{sym}	+0.4	+0.4	+0.4	+0.4
$\Delta G^{\circ}_{\text{bind,total}}$	-21.9 ± 0.9	-3.4 ± 0.4	+2.7 ± 0.8	-10.6 ± 1.1
$\Delta G^{\circ}_{\text{bind,total}}/helix$	-11.0 ± 0.4	-1.7 ± 0.2	+1.3 ± 0.4	-5.3 ± 0.5

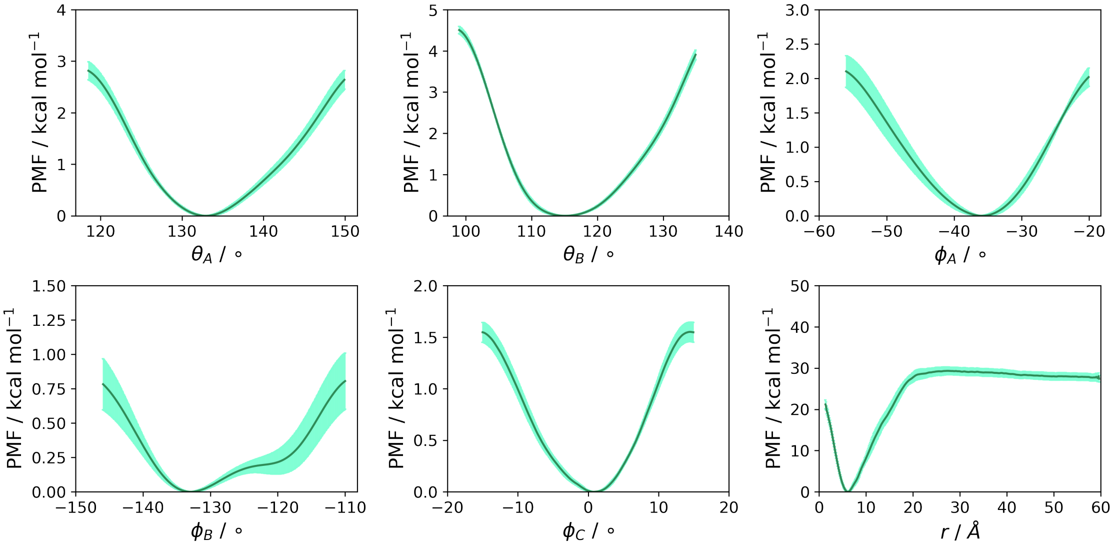

Figure S15: Individual PMF profiles for CC-Di. The shaded areas represent standard error from independent simulation replicates. Simulations were carried out with the first set of anchor points (see Table S3).

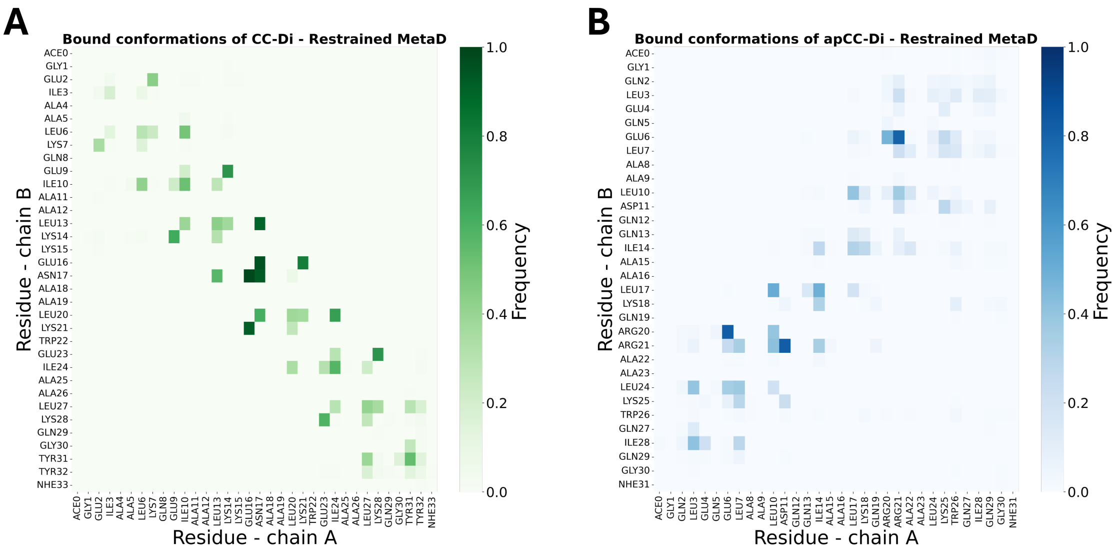

Figure S16: Intermolecular interactions between chains A and B of the bound conformations of **A**. CC-Di and **B**. apCC-Di from metadynamics simulations with Boresch restraints. Interactions characteristic of coiled-coil dimers are rescued in the presence of restraints.

Table S3: Free energy contributions for the experimental and designed CC-Di simulations. Values in kcal mol^{-1}. The anchor points for set 1 are the Cα atoms of residues c = Ile 11, b = Lys 16, a = Asn 18, A = Asn 52, B = Trp 57, C = Ile 59. The anchor points for set 2 are the Cα atoms of residues c = Leu 14, b = Asn 18, a = Lys 16, A = Lys 50, B = Asn 52, C = Leu 48.

	**CC-Di**		
	**Set 1 Anchor Points**		**Set 2 Anchor Points**
**Contribution**	**Experimental Structure**	**ISAMBARD Design**	**Experimental Structure**
ΔG_{θ_A}	-0.22 ± 0.01	-0.28 ± 0.04	-0.09 ± 0.01
ΔG_{θ_B}	-0.23 ± 0.01	-0.23 ± 0.04	-0.09 ± 0.01
ΔG_{ϕ_A}	-0.30 ± 0.02	-0.30 ± 0.01	-0.36 ± 0.06
ΔG_{ϕ_B}	-0.54 ± 0.04	-0.47 ± 0.02	-0.20 ± 0.01
ΔG_{ϕ_C}	-0.28 ± 0.01	-0.26 ± 0.01	-0.22 ± 0.01
$\Delta G^{\circ}_{\text{sep}}$	-27.6 ± 0.9	-27.4 ± 0.5	-28.5 ± 0.3
$\Delta G^{\circ}_{\text{corr}}$	+6.8 ± 0.1	+6.8 ± 0.1	+7.3 ± 0.1
ΔG_{sym}	+0.4	+0.4	+0.4
$\Delta G^{\circ}_{\text{bind,total}}$	-21.9 ± 0.9	-21.7 ± 0.5	-21.8 ± 0.3
$\Delta G^{\circ}_{\text{bind,total}}/helix$	-11.0 ± 0.4	-10.9 ± 0.3	-10.9 ± 0.1

Table S4: Free energy contributions for the CC-Tri simulations, with and without RMSD restraints. Only the contributions from the trimer dissociation step are shown. Values in kcal mol^{-1}.

	**CC-Tri**	
**Contribution**	**w/o RMSD restraints**	**w/ RMSD restraints**
ΔG_{θ_A}	-0.25 ± 0.01	-0.30 ± 0.02
ΔG_{θ_B}	-0.22 ± 0.01	-0.23 ± 0.02
ΔG_{ϕ_A}	-0.50 ± 0.02	-0.32 ± 0.01
ΔG_{ϕ_B}	-0.32 ± 0.02	-0.29 ± 0.02
ΔG_{ϕ_C}	-0.23 ± 0.01	-0.35 ± 0.01
$\Delta G_{RMSD_{bound}} + \Delta G_{RMSD_{unbound}}$	-	+6.5 ± 1.7
$\Delta G^{\circ}_{\mathrm{sep}}$	-31.6 ± 1.5	-37.6 ± 1.6
$\Delta G^{\circ}_{\mathrm{corr}}$	+7.0 ± 0.1	+7.7 ± 0.1
$\Delta G^{\circ}_{\mathrm{bind}}$	-26.1 ± 1.5	-24.9 ± 2.3

Table S5: Free energy contributions for the CC-Tri simulations with RMSD restraints. The contributions from the trimer and dimer steps are summed to yield the $\Delta G^{\circ}_{\mathrm{bind,total}}/helix$ value for the whole complex. Values in kcal mol^{-1}.

	**CC-Tri**	
**Contribution**	**Trimer Step**	**Dimer Step**
ΔG_{θ_A}	-0.30 ± 0.02	-0.53 ± 0.03
ΔG_{θ_B}	-0.23 ± 0.02	-0.11 ± 0.01
ΔG_{ϕ_A}	-0.32 ± 0.01	-0.56 ± 0.01
ΔG_{ϕ_B}	-0.29 ± 0.02	-0.20 ± 0.01
ΔG_{ϕ_C}	-0.35 ± 0.01	-0.47 ± 0.01
$\Delta G_{RMSD_{bound}} + \Delta G_{RMSD_{unbound}}$	+6.5 ± 1.7	+0.9 ± 0.8
$\Delta G^{\circ}_{\mathrm{sep}}$	-37.6 ± 1.6	-24.3 ± 0.9
$\Delta G^{\circ}_{\mathrm{corr}}$	+7.7 ± 0.1	+9.1 ± 0.1
$\Delta G^{\circ}_{\mathrm{bind}}$	-24.9 ± 2.3	-16.2 ± 1.2
ΔG_{sym}	+1.1	
$\Delta G^{\circ}_{\mathrm{bind,total}}$	-40.0 ± 2.6	
$\Delta G^{\circ}_{\mathrm{bind,total}}/helix$	-13.3 ± 0.9	

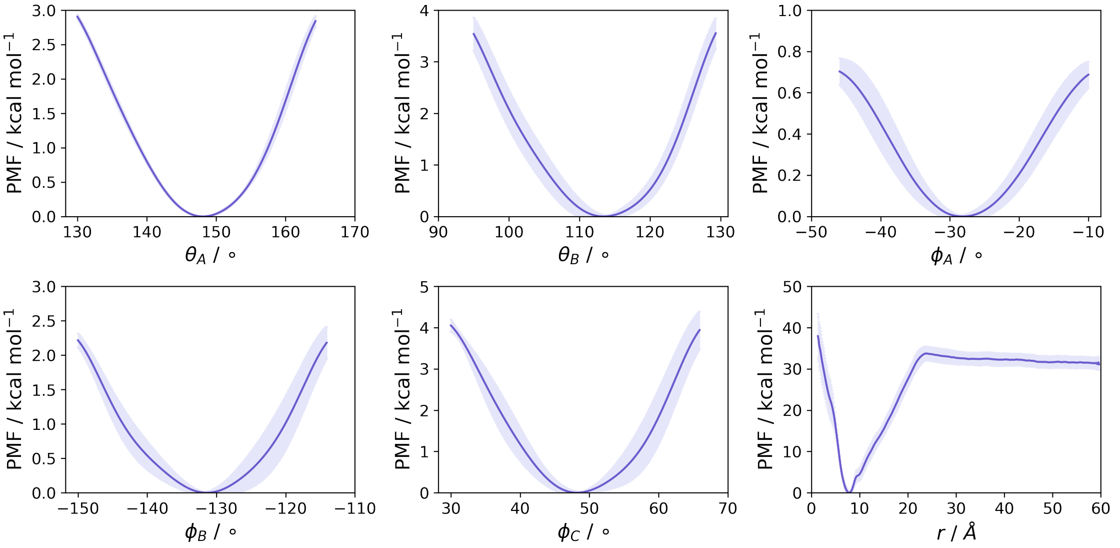

Figure S17: Individual PMF profiles for CC-Tri (trimer separation), without RMSD restraints on the 'receptor' helices. The shaded areas represent standard error from independent simulation replicates.

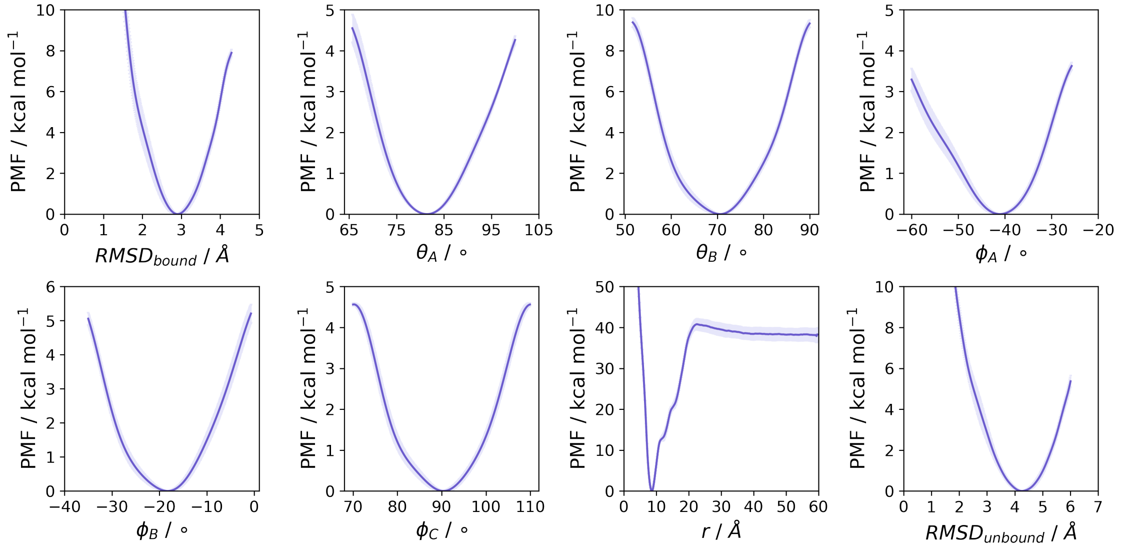

Figure S18: Individual PMF profiles for CC-Tri (trimer separation), with RMSD restraints on the 'receptor' helices. The shaded areas represent standard error from independent simulation replicates.

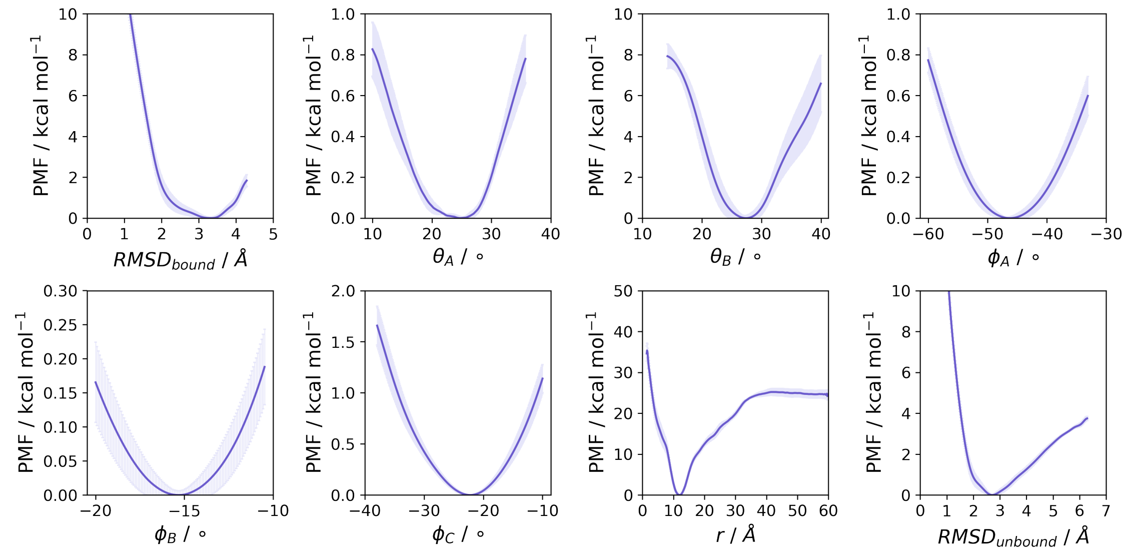

Figure S19: Individual PMF profiles for CC-Tri (dimer separation). The shaded areas represent standard error from independent simulation replicates.

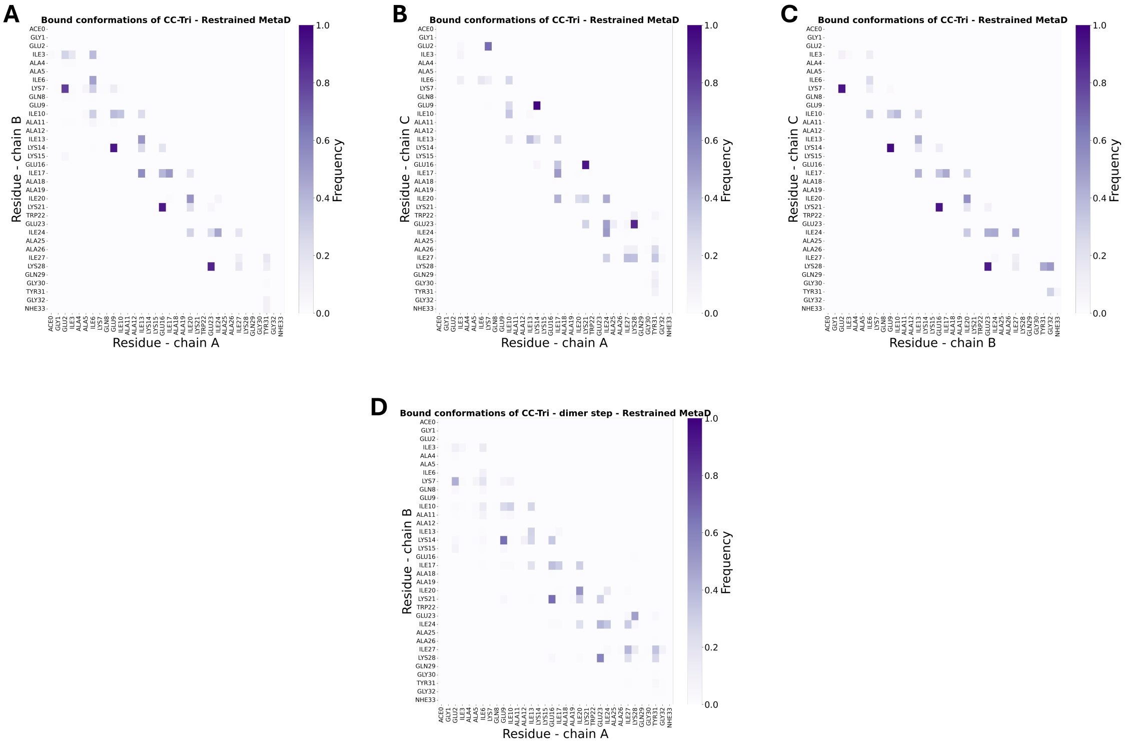

Figure S20: Intermolecular interactions between pairs of chains of the bound conformations of the CC-Tri complex from metadynamics simulations with helical, RMSD and Boresch restraints. **A-C**. Interactions between chains A-B, A-C and B-C in the trimer step. **D**. Interactions between chains B and C in the dimer step (chains have been renamed to A and B respectively as only two chains are present in the dimer structure). The restraints are effectively restricting the 'receptor' helices B-C to their 'open' conformation at both trimer and dimer dissociation steps.

Table S6: Free energy contributions from the up-down-up CC-Tri, separated with scheme 1 (helix A removed first, followed by helix B). Values in kcal mol^{-1}.

	**Up-Down-Up CC-Tri - Scheme 1**	
**Contribution**	**Trimer Step**	**Dimer Step**
ΔG_{θ_A}	-0.22 ± 0.01	-0.31 ± 0.06
ΔG_{θ_B}	-0.13 ± 0.01	-0.30 ± 0.05
ΔG_{ϕ_A}	-0.57 ± 0.03	-0.46 ± 0.01
ΔG_{ϕ_B}	-0.37 ± 0.02	-0.37 ± 0.01
ΔG_{ϕ_C}	-0.38 ± 0.02	-0.34 ± 0.03
$\Delta G_{RMSD_{bound}} + \Delta G_{RMSD_{unbound}}$	-0.9 ± 3.3	-4.3 ± 2.3
$\Delta G^{\circ}_{\text{sep}}$	-11.8 ± 1.3	-19.0 ± 1.5
$\Delta G^{\circ}_{\text{corr}}$	+8.8 ± 0.1	+8.3 ± 0.1
$\Delta G^{\circ}_{\text{bind}}$	-5.5 ± 3.5	-16.8 ± 2.7
ΔG_{sym}	+1.1	
$\Delta G^{\circ}_{\text{bind,total}}$	-21.3 ± 4.5	
$\Delta G^{\circ}_{\text{bind,total}}/helix$	-7.1 ± 1.5	

Table S7: Free energy contributions from the up-down-up CC-Tri, separated with scheme 2 (helix B removed first, followed by helix A). Values in kcal mol^{-1}.

	**Up-Down-Up CC-Tri - Scheme 2**	
**Contribution**	**Trimer Step**	**Dimer Step**
ΔG_{θ_A}	-0.20 ± 0.02	-0.45 ± 0.04
ΔG_{θ_B}	-0.22 ± 0.01	-0.26 ± 0.02
ΔG_{ϕ_A}	-0.35 ± 0.01	-0.42 ± 0.01
ΔG_{ϕ_B}	-0.32 ± 0.01	-0.54 ± 0.01
ΔG_{ϕ_C}	-0.41 ± 0.05	-0.40 ± 0.03
$\Delta G_{RMSD_{bound}} + \Delta G_{RMSD_{unbound}}$	+1.1 ± 2.1	-0.6 ± 1.0
$\Delta G^{\circ}_{\text{sep}}$	-15.0 ± 0.9	-21.7 ± 1.7
$\Delta G^{\circ}_{\text{corr}}$	+8.3 ± 0.1	+8.6 ± 0.1
$\Delta G^{\circ}_{\text{bind}}$	-7.1 ± 2.3	-15.8 ± 2.0
ΔG_{sym}	+1.1	
$\Delta G^{\circ}_{\text{bind,total}}$	-21.8 ± 3.0	
$\Delta G^{\circ}_{\text{bind,total}}/helix$	-7.2 ± 1.0	

Table S8: Free energy contributions for the trimeric CC-Di complex. Values in kcal mol^{-1}.

	**Trimeric CC-Di**	
**Contribution**	**Trimer Step**	**Dimer Step**
ΔG_{θ_A}	-0.36 ± 0.02	-0.23 ± 0.01
ΔG_{θ_B}	-0.17 ± 0.01	-0.23 ± 0.03
ΔG_{ϕ_A}	-0.56 ± 0.04	-0.48 ± 0.01
ΔG_{ϕ_B}	-0.35 ± 0.01	-0.35 ± 0.01
ΔG_{ϕ_C}	-0.17 ± 0.01	-0.30 ± 0.01
$\Delta G_{RMSD_{bound}} + \Delta G_{RMSD_{unbound}}$	+11.6 ± 2.0	+0.2 ± 1.9
$\Delta G^{\circ}_{\text{sep}}$	-28.4 ± 2.2	-22.3 ± 0.4
$\Delta G^{\circ}_{\text{corr}}$	+8.4 ± 0.1	+8.3 ± 0.1
$\Delta G^{\circ}_{\text{bind}}$	-10.0 ± 3.0	-15.3 ± 2.0
ΔG_{sym}	+1.1	
$\Delta G^{\circ}_{\text{bind,total}}$	-24.3 ± 3.6	
$\Delta G^{\circ}_{\text{bind,total}}/helix$	-8.1 ± 1.2	

Table S9: Free energy contributions for the tetrameric CC-Di complex. Values in kcal mol^{-1}.

	**Tetrameric CC-Di**		
**Contribution**	**Tetramer Step**	**Trimer Step**	**Dimer Step**
ΔG_{θ_A}	-0.32 ± 0.08	-0.32 ± 0.03	-0.23 ± 0.01
ΔG_{θ_B}	-0.13 ± 0.01	-0.18 ± 0.01	-0.17 ± 0.01
ΔG_{ϕ_A}	-0.28 ± 0.01	-0.32 ± 0.02	-0.01 ± 0.01
ΔG_{ϕ_B}	-0.26 ± 0.01	-0.28 ± 0.01	-0.20 ± 0.01
ΔG_{ϕ_C}	-0.09 ± 0.01	-0.44 ± 0.05	-0.18 ± 0.01
$\Delta G_{RMSD_{bound}} + \Delta G_{RMSD_{unbound}}$	+14.2 ± 1.5	+12.0 ± 2.2	+1.2 ± 1.3
$\Delta G^{\circ}_{\text{sep}}$	-30.3 ± 1.2	-21.6 ± 0.7	-21.3 ± 1.2
$\Delta G^{\circ}_{\text{corr}}$	+8.2 ± 0.1	+8.2 ± 0.1	+8.2 ± 0.1
$\Delta G^{\circ}_{\text{bind}}$	-8.9 ± 1.9	-2.9 ± 2.3	-12.7 ± 1.8
ΔG_{sym}	+1.9		
$\Delta G^{\circ}_{\text{bind,total}}$	-22.6 ± 3.5		
$\Delta G^{\circ}_{\text{bind,total}}/helix$	-5.6 ± 0.9		

Table S10: Free energy contributions for the dimeric CC-Tri complex. Values in kcal mol^{-1}.

**Contribution**	**Dimeric CC-Tri**
ΔG_{θ_A}	-0.31 ± 0.02
ΔG_{θ_B}	-0.18 ± 0.01
ΔG_{ϕ_A}	-0.58 ± 0.01
ΔG_{ϕ_B}	-0.48 ± 0.01
ΔG_{ϕ_C}	-0.50 ± 0.01
$\Delta G^{\circ}_{\text{sep}}$	-24.7 ± 1.0
$\Delta G^{\circ}_{\text{corr}}$	+8.5 ± 0.1
ΔG_{sym}	+0.4
$\Delta G^{\circ}_{\text{bind,total}}$	-17.9 ± 1.0
$\Delta G^{\circ}_{\text{bind,total}}/helix$	-9.0 ± 0.5

Table S11: Free energy contributions for the tetrameric CC-Tri complex. Values in kcal mol^{-1}.

	**Tetrameric CC-Tri**		
**Contribution**	**Tetramer Step**	**Trimer Step**	**Dimer Step**
ΔG_{θ_A}	-0.36 ± 0.02	-0.32 ± 0.03	-0.29 ± 0.01
ΔG_{θ_B}	-0.20 ± 0.01	-0.17 ± 0.02	-0.15 ± 0.02
ΔG_{ϕ_A}	-0.41 ± 0.02	-0.32 ± 0.02	-0.41 ± 0.02
ΔG_{ϕ_B}	-0.21 ± 0.02	-0.23 ± 0.03	-0.28 ± 0.01
ΔG_{ϕ_C}	-0.39 ± 0.02	-0.02 ± 0.01	-0.01 ± 0.01
$\Delta G_{RMSD_{bound}} + \Delta G_{RMSD_{unbound}}$	+24.8 ± 2.0	+2.1 ± 1.8	-0.2 ± 0.9
$\Delta G^{\circ}_{\text{sep}}$	-16.4 ± 1.4	-32.9 ± 0.7	-22.9 ± 0.9
$\Delta G^{\circ}_{\text{corr}}$	+8.2 ± 0.1	+8.2 ± 0.1	+8.2 ± 0.1
$\Delta G^{\circ}_{\text{bind}}$	+15.0 ± 2.5	-23.6 ± 2.0	-16.0 ± 1.3
ΔG_{sym}	+1.9		
$\Delta G^{\circ}_{\text{bind,total}}$	-22.8 ± 3.4		
$\Delta G^{\circ}_{\text{bind,total}}/helix$	-5.7 ± 0.9		

Table S12: Free energy contributions for the dimeric CC-Tet complex. Values in kcal mol^{-1}.

**Contribution**	**Dimeric CC-Tet**
ΔG_{θ_A}	-0.28 ± 0.01
ΔG_{θ_B}	-0.29 ± 0.01
ΔG_{ϕ_A}	-0.33 ± 0.01
ΔG_{ϕ_B}	-0.02 ± 0.01
ΔG_{ϕ_C}	-0.30 ± 0.02
$\Delta G^{\circ}_{\text{sep}}$	-26.4 ± 1.6
$\Delta G^{\circ}_{\text{corr}}$	+8.2 ± 0.1
ΔG_{sym}	+0.4
$\Delta G^{\circ}_{\text{bind,total}}$	-19.1 ± 1.6
$\Delta G^{\circ}_{\text{bind,total}}/helix$	-9.5 ± 0.8

Table S13: Free energy contributions for the trimeric CC-Tet complex. Values in kcal mol^{-1}.

	**Trimeric CC-Tet**	
**Contribution**	**Trimer Step**	**Dimer Step**
ΔG_{θ_A}	-0.19 ± 0.01	-0.33 ± 0.03
ΔG_{θ_B}	-0.16 ± 0.01	-0.22 ± 0.01
ΔG_{ϕ_A}	-0.25 ± 0.01	-0.01 ± 0.01
ΔG_{ϕ_B}	-0.22 ± 0.01	-0.01 ± 0.01
ΔG_{ϕ_C}	-0.30 ± 0.01	-0.31 ± 0.01
$\Delta G_{RMSD_{bound}} + \Delta G_{RMSD_{unbound}}$	+11.9 ± 2.4	+3.4 ± 0.4
$\Delta G^{\circ}_{\text{sep}}$	-30.6 ± 1.2	-24.3 ± 1.0
$\Delta G^{\circ}_{\text{corr}}$	+8.3 ± 0.1	+8.2 ± 0.1
$\Delta G^{\circ}_{\text{bind}}$	-11.5 ± 2.7	-13.6 ± 1.0
ΔG_{sym}	+1.1	
$\Delta G^{\circ}_{\text{bind,total}}$	-24.1 ± 2.9	
$\Delta G^{\circ}_{\text{bind,total}}/helix$	-8.0 ± 1.0	

Table S14: Free energy contributions for CC-Tet. Values in kcal mol^{-1}.

	CC-Tet		
**Contribution**	**Tetramer Step**	**Trimer Step**	**Dimer Step**
ΔG_{θ_A}	-0.16 ± 0.01	-0.26 ± 0.01	-0.25 ± 0.02
ΔG_{θ_B}	-0.10 ± 0.01	-0.20 ± 0.01	-0.20 ± 0.01
ΔG_{ϕ_A}	-0.01 ± 0.01	-0.32 ± 0.01	-0.19 ± 0.01
ΔG_{ϕ_B}	-0.19 ± 0.01	-0.34 ± 0.01	-0.47 ± 0.01
ΔG_{ϕ_C}	-0.06 ± 0.01	-0.20 ± 0.02	-0.29 ± 0.01
$\Delta G_{RMSD_{bound}} + \Delta G_{RMSD_{unbound}}$	+29.2 ± 1.2	+4.1 ± 1.5	-3.7 ± 0.7
$\Delta G^{\circ}_{\text{sep}}$	-39.7 ± 2.2	-39.5 ± 1.9	-20.3 ± 1.4
$\Delta G^{\circ}_{\text{corr}}$	+8.2 ± 0.1	+8.2 ± 0.1	+7.1 ± 0.1
$\Delta G^{\circ}_{\text{bind}}$	-2.8 ± 2.5	-28.6 ± 2.4	-18.2 ± 1.6
ΔG_{sym}	+1.9		
$\Delta G^{\circ}_{\text{bind,total}}$	-47.6 ± 3.9		
$\Delta G^{\circ}_{\text{bind,total}}/helix$	-11.9 ± 1.0		

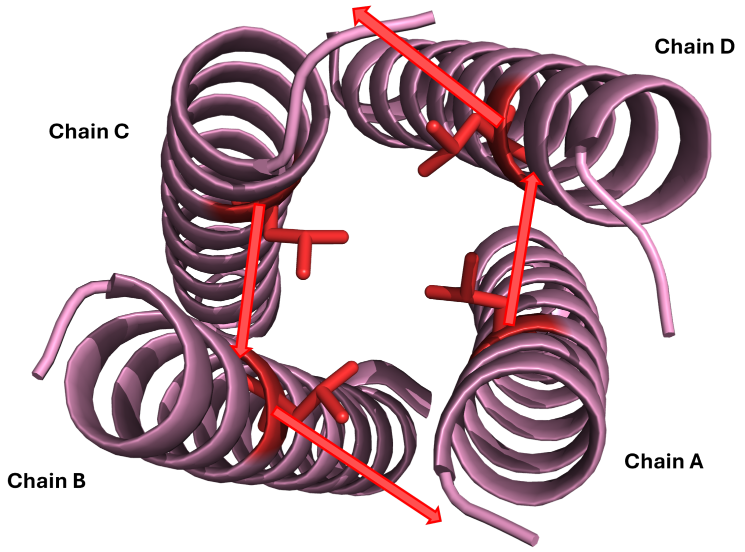

Figure S21: The two different types of packing angle at the a position of the heptad repeat of CC-Tet. A layer of Leu residues at the a position is shown in red sticks. The red arrows represent the Cα-Cβ bond vector of the Leu residues. It can be seen that the vector points perpendicularly towards the neighbouring helix in the A-D and B-C interfaces, whereas it points away from the neighbouring helix in the A-B and C-D interfaces, conferring structural asymmetry.

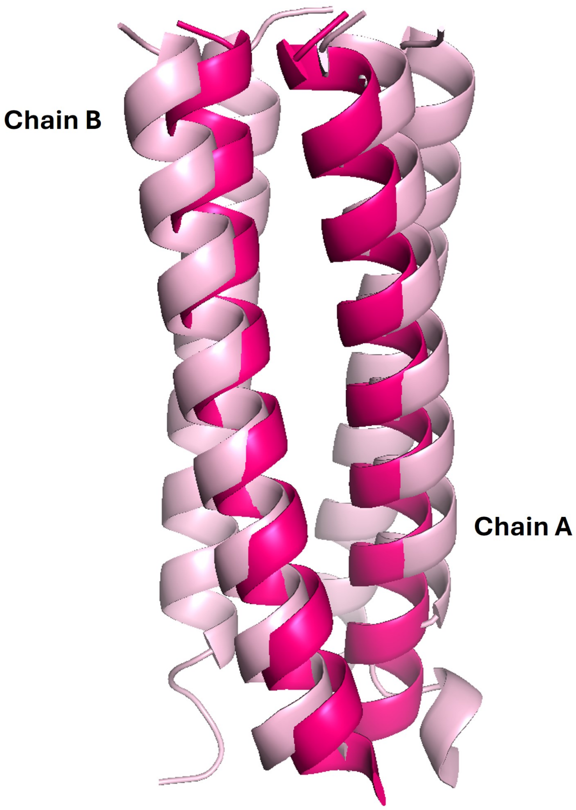

Figure S22: The X-ray structure of CC-Tet (light pink) aligned with the designed structure of the dimeric CC-Tet (dark pink). CC-Tet possesses two distinct types of interfaces, where helices A-B and C-D have the same type of interface, and helices A-D and B-C have a different type of interface.[38] The dimeric CC-Tet aligns on the A-B chains of CC-Tet. The RMSD alignment is 2.5 Å. Rendered with PyMOL.[5]

Table S15: Free energy contributions for the CC-Hex*-L24E tetramer at low pH. Values in kcal mol^{-1}.

	**L24E Tetramer - Low pH**		
**Contribution**	**Tetramer Step**	**Trimer Step**	**Dimer Step**
ΔG_{θ_A}	-0.26 ± 0.01	-0.51 ± 0.06	-0.37 ± 0.01
ΔG_{θ_B}	-0.29 ± 0.01	-0.44 ± 0.04	-0.22 ± 0.01
ΔG_{ϕ_A}	-0.35 ± 0.01	-0.52 ± 0.04	-0.33 ± 0.01
ΔG_{ϕ_B}	-0.40 ± 0.01	-0.51 ± 0.06	-0.52 ± 0.02
ΔG_{ϕ_C}	-0.48 ± 0.02	-0.35 ± 0.01	-0.02 ± 0.01
$\Delta G_{RMSD_{bound}} + \Delta G_{RMSD_{unbound}}$	+14.0 ± 1.0	+2.3 ± 1.6	+0.1 ± 0.7
$\Delta G^{\circ}_{\text{sep}}$	-23.3 ± 0.9	-12.0 ± 0.8	-9.8 ± 0.8
$\Delta G^{\circ}_{\text{corr}}$	+8.4 ± 0.1	+8.2 ± 0.1	+8.3 ± 0.1
$\Delta G^{\circ}_{\text{bind}}$	-2.7 ± 1.4	-3.9 ± 1.8	-2.9 ± 1.1
ΔG_{sym}	+1.9		
$\Delta G^{\circ}_{\text{bind,total}}$	-7.6 ± 2.5		
$\Delta G^{\circ}_{\text{bind,total}}/helix$	-1.9 ± 0.6		

Table S16: Free energy contributions for the CC-Hex*-L24E tetramer at high pH. Values in kcal mol^{-1}.

	**L24E Tetramer - High pH**		
**Contribution**	**Tetramer Step**	**Trimer Step**	**Dimer Step**
ΔG_{θ_A}	-0.23 ± 0.01	-0.41 ± 0.02	-0.41 ± 0.01
ΔG_{θ_B}	-0.38 ± 0.01	-0.33 ± 0.03	-0.35 ± 0.06
ΔG_{ϕ_A}	-0.35 ± 0.04	-0.48 ± 0.03	-0.35 ± 0.01
ΔG_{ϕ_B}	-0.36 ± 0.01	-0.50 ± 0.01	-0.01 ± 0.01
ΔG_{ϕ_C}	-0.33 ± 0.01	-0.40 ± 0.01	-0.02 ± 0.01
$\Delta G_{RMSD_{bound}} + \Delta G_{RMSD_{unbound}}$	+5.6 ± 1.6	-4.6 ± 3.4	+2.2 ± 1.0
$\Delta G^{\circ}_{\text{sep}}$	-23.3 ± 1.8	-12.3 ± 1.1	-11.1 ± 1.0
$\Delta G^{\circ}_{\text{corr}}$	+8.4 ± 0.1	+8.1 ± 0.1	+8.3 ± 0.1
$\Delta G^{\circ}_{\text{bind}}$	-10.9 ± 2.5	-10.9 ± 3.5	-1.7 ± 1.4
ΔG_{sym}	+1.9		
$\Delta G^{\circ}_{\text{bind,total}}$	-21.7 ± 4.5		
$\Delta G^{\circ}_{\text{bind,total}}/helix$	-5.4 ± 1.1		

Table S17: Free energy contributions for the CC-Hex*-L24E hexamer at low pH. Values in kcal mol^{-1}.

	**L24E Hexamer - Low pH**		
**Contribution**	**Hexamer Step**	**Pentamer Step**	**Tetramer Step**
ΔG_{θ_A}	-0.23 ± 0.02	-0.17 ± 0.01	-0.52 ± 0.04
ΔG_{θ_B}	-0.18 ± 0.01	-0.23 ± 0.01	-0.10 ± 0.01
ΔG_{ϕ_A}	-0.19 ± 0.01	-0.30 ± 0.01	-0.27 ± 0.04
ΔG_{ϕ_B}	-0.26 ± 0.01	-0.33 ± 0.01	-0.43 ± 0.02
ΔG_{ϕ_C}	-0.24 ± 0.01	-0.25 ± 0.03	-0.02 ± 0.01
$\Delta G_{RMSD_{bound}} + \Delta G_{RMSD_{unbound}}$	-8.3 ± 6.7	-7.1 ± 2.4	+7.7 ± 1.5
$\Delta G^{\circ}_{\text{sep}}$	-26.1 ± 1.0	-15.5 ± 1.4	-9.2 ± 1.0
$\Delta G^{\circ}_{\text{corr}}$	+8.2 ± 0.1	+8.2 ± 0.1	+8.6 ± 0.1
$\Delta G^{\circ}_{\text{bind}}$	-27.4 ± 6.7	-15.7 ± 2.8	+5.8 ± 1.8

**Contribution**	**Trimer Step**	**Dimer Step**
ΔG_{θ_A}	-0.37 ± 0.01	-0.35 ± 0.01
ΔG_{θ_B}	-0.18 ± 0.03	-0.22 ± 0.07
ΔG_{ϕ_A}	-0.73 ± 0.01	-0.31 ± 0.03
ΔG_{ϕ_B}	-0.40 ± 0.01	-0.23 ± 0.02
ΔG_{ϕ_C}	-0.02 ± 0.01	-0.02 ± 0.01
$\Delta G_{RMSD_{bound}} + \Delta G_{RMSD_{unbound}}$	+5.0 ± 1.0	+4.3 ± 1.1
$\Delta G^{\circ}_{\text{sep}}$	-12.5 ± 1.1	-9.7 ± 0.7
$\Delta G^{\circ}_{\text{corr}}$	+8.6 ± 0.1	+8.6 ± 0.1
$\Delta G^{\circ}_{\text{bind}}$	-0.6 ± 1.5	+2.1 ± 1.3

ΔG_{sym}	+3.9
$\Delta G^{\circ}_{\text{bind,total}}$	-31.9 ± 7.8
$\Delta G^{\circ}_{\text{bind,total}}/helix$	-5.3 ± 1.3

Table S18: Free energy contributions for the CC-Hex*-L24E hexamer at high pH. Values in kcal mol^{-1}.

	**L24E Hexamer - High pH**		
**Contribution**	**Hexamer Step**	**Pentamer Step**	**Tetramer Step**
ΔG_{θ_A}	-0.24 ± 0.04	-0.37 ± 0.03	-0.32 ± 0.05
ΔG_{θ_B}	-0.05 ± 0.01	-0.22 ± 0.02	-0.16 ± 0.03
ΔG_{ϕ_A}	-0.18 ± 0.01	-0.32 ± 0.03	-0.27 ± 0.01
ΔG_{ϕ_B}	-0.19 ± 0.01	-0.49 ± 0.04	-0.43 ± 0.06
ΔG_{ϕ_C}	-0.26 ± 0.01	-0.25 ± 0.03	-0.02 ± 0.01
$\Delta G_{RMSD_{bound}} + \Delta G_{RMSD_{unbound}}$	+8.4 ± 1.3	+24.3 ± 7.8	-6.3 ± 4.2
$\Delta G^{\circ}_{\text{sep}}$	-7.6 ± 0.3	-15.3 ± 2.9	-16.3 ± 1.9
$\Delta G^{\circ}_{\text{corr}}$	+8.5 ± 0.1	+8.2 ± 0.1	+8.4 ± 0.1
$\Delta G^{\circ}_{\text{bind}}$	+8.4 ± 1.3	+15.5 ± 8.3	-15.4 ± 4.6

**Contribution**	**Trimer Step**	**Dimer Step**
ΔG_{θ_A}	-0.42 ± 0.01	-0.38 ± 0.03
ΔG_{θ_B}	-0.22 ± 0.02	-0.09 ± 0.02
ΔG_{ϕ_A}	-0.53 ± 0.03	-0.37 ± 0.02
ΔG_{ϕ_B}	-0.51 ± 0.03	-0.45 ± 0.01
ΔG_{ϕ_C}	-0.29 ± 0.01	-0.02 ± 0.01
$\Delta G_{RMSD_{bound}} + \Delta G_{RMSD_{unbound}}$	-4.7 ± 4.0	-1.1 ± 0.4
$\Delta G^{\circ}_{\text{sep}}$	-12.5 ± 1.3	-11.5 ± 0.7
$\Delta G^{\circ}_{\text{corr}}$	+8.2 ± 0.1	+8.5 ± 0.1
$\Delta G^{\circ}_{\text{bind}}$	-11.0 ± 4.2	-5.4 ± 0.8

ΔG_{sym}	+3.9
$\Delta G^{\circ}_{\text{bind,total}}$	-4.0 ± 10.5
$\Delta G^{\circ}_{\text{bind,total}}/helix$	-0.7 ± 1.8

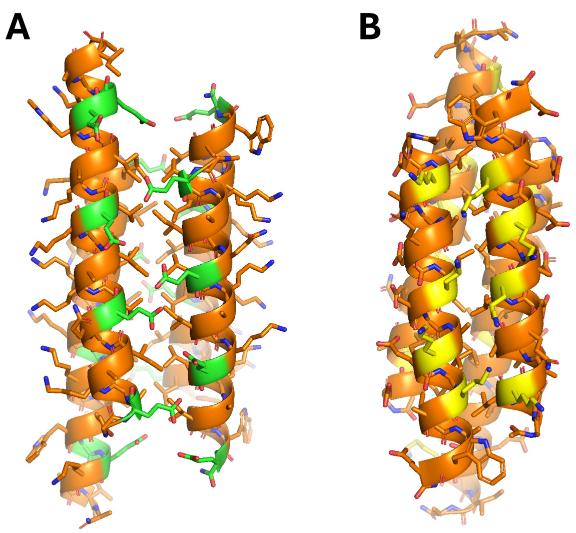

Figure S23: The X-ray structure of the CC-Hex*-L24E antiparallel tetramer shows structural asymmetry due to the presence of two distinct interfaces. **A**. The 'wide' interface formed between chains A-D and B-C, flanked by Glu residues (shown in green). **B**. The 'narrow' interface formed between chains A-B and C-D, flanked by Lys residues (shown in yellow). Rendered with PyMOL.[5]

References

(1) Duboué-Dijon, E.; Hénin, J. Building intuition for binding free energy calculations: Bound state definition, restraints, and symmetry. *J. Chem. Phys.* **2021**, *154*, 204101.

(2) De Jong, D. H.; Schäfer, L. V.; De Vries, A. H.; Marrink, S. J.; Berendsen, H. J. C.; Grubmüller, H. Determining equilibrium constants for dimerization reactions from molecular dynamics simulations. *J. Comput. Chem.* **2011**, *32*, 1919–1928.

(3) Jost Lopez, A.; Quoika, P. K.; Linke, M.; Hummer, G.; Köfinger, J. Quantifying Protein–Protein Interactions in Molecular Simulations. *J. Phys. Chem. B* **2020**, *124*, 4673–4685.

(4) Wood, C. W.; Heal, J. W.; Thomson, A. R.; Bartlett, G. J.; Ibarra, A.; Brady, R. L.; Sessions, R. B.; Woolfson, D. N. ISAMBARD: An open-source computational environment for biomolecular analysis, modelling and design. *Bioinformatics* **2017**, *33*, 3043–3050.

(5) Schrödinger, LLC PyMOL: The PyMOL Molecular Graphics System, Version 1.8, Schrödinger, LLC.

(6) Steinmetz, M. O.; Jelesarov, I.; Matousek, W. M.; Honnappa, S.; Jahnke, W.; Missimer, J. H.; Frank, S.; Alexandrescu, A. T.; Kammerer, R. A. Molecular basis of coiled-coil formation. *PNAS* **2007**, *104*, 7062–7067.

(7) Nguyen, H.; Roe, D. R.; Simmerling, C. Improved Generalized Born Solvent Model Parameters for Protein Simulations. *J. Chem. Theory Comput.* **2013**, *9*, 2020–2034.

(8) Nguyen, H.; Maier, J.; Huang, H.; Perrone, V.; Simmerling, C. Folding Simulations for Proteins with Diverse Topologies Are Accessible in Days with a Physics-Based Force Field and Implicit Solvent. *JACS* **2014**, *136*, 13959–13962.

(9) Shao, Q.; Zhu, W. Assessing AMBER force fields for protein folding in an implicit solvent. *Phys. Chem. Chem. Phys.* **2018**, *20*, 7206–7216.

(10) Cornell, W. D.; Cieplak, P.; Bayly, C. I.; Gould, I. R.; Merz, K. M.; Ferguson, D. M.; Spellmeyer, D. C.; Fox, T.; Caldwell, J. W.; Kollman, P. A. A Second Generation Force Field for the Simulation of Proteins, Nucleic Acids, and Organic Molecules. *JACS* **1995**, *117*, 5179–5197.

(11) Kollman, P.; Dixon, R.; Cornell, W.; Fox, T.; Chipot, C.; Pohorille, A. *Computer Simulation of Biomolecular Systems*; Springer Netherlands: Dordrecht, 1997; pp 83–96.

(12) Cheatham, T. E.; Cieplak, P.; Kollman, P. A. A Modified Version of the Cornell *et al.* Force Field with Improved Sugar Pucker Phases and Helical Repeat. *J. Biomol. Struct. Dyn.* **1999**, *16*, 845–862.

(13) Hornak, V.; Abel, R.; Okur, A.; Strockbine, B.; Roitberg, A.; Simmerling, C. Comparison of multiple Amber force fields and development of improved protein backbone parameters. *Proteins: Struct., Funct., Bioinf.* **2006**, *65*, 712–725.

(14) Li, D.; Brüschweiler, R. NMR-Based Protein Potentials. *Angew. Chem., Int. Ed. Engl.* **2010**, *49*, 6778–6780.

(15) Maier, J. A.; Martinez, C.; Kasavajhala, K.; Wickstrom, L.; Hauser, K. E.; Simmerling, C. ff14SB: Improving the Accuracy of Protein Side Chain and Backbone Parameters from ff99SB. *J. Chem. Theory Comput.* **2015**, *11*, 3696–3713.

(16) Onufriev, A.; Bashford, D.; Case, D. A. Exploring protein native states and large-scale conformational changes with a modified generalized born model. *Proteins: Struct., Funct., Bioinf.* **2004**, *55*, 383–394.

(17) Mongan, J.; Simmerling, C.; McCammon, J. A.; Case, D. A.; Onufriev, A. Generalized Born Model with a Simple, Robust Molecular Volume Correction. *J. Chem. Theory Comput.* **2007**, *3*, 156–169.

(18) Case, D. A. et al. AmberTools. *J. Chem. Inf. Model.* **2023**, *63*, 6183–6191.

(19) Eastman, P.; Swails, J.; Chodera, J. D.; McGibbon, R. T.; Zhao, Y.; Beauchamp, K. A.; Wang, L.-P.; Simmonett, A. C.; Harrigan, M. P.; Stern, C. D.; Wiewiora, R. P.; Brooks, B. R.; Pande, V. S. OpenMM 7: Rapid development of high performance algorithms for molecular dynamics. *PLoS Comput. Biol.* **2017**, *13*, e1005659.

(20) McGibbon, R. T.; Beauchamp, K. A.; Harrigan, M. P.; Klein, C.; Swails, J. M.; Hernández, C. X.; Schwantes, C. R.; Wang, L.-P.; Lane, T. J.; Pande, V. S. MDTraj: A Modern Open Library for the Analysis of Molecular Dynamics Trajectories. *Biophys. J.* **2015**, *109*, 1528–1532.

(21) Meisenberg, G.; Simmons, W. H. *Principles of Medical Biochemistry*, 4th ed.; Saunders, 2017; Chapter 2, pp 18–32.

(22) Maffucci, I.; Contini, A. An Updated Test of AMBER Force Fields and Implicit Solvent Models in Predicting the Secondary Structure of Helical, β-Hairpin, and Intrinsically Disordered Peptides. *J. Chem. Theory Comput.* **2016**, *12*, 714–727.

(23) Robinson, M. K.; Monroe, J. I.; Shell, M. S. Are AMBER Force Fields and Implicit Solvation Models Additive? A Folding Study with a Balanced Peptide Test Set. *J. Chem. Theory Comput.* **2016**, *12*, 5631–5642.

(24) Lang, E. J. M.; Baker, E. G.; Woolfson, D. N.; Mulholland, A. J. Generalized Born Implicit Solvent Models Do Not Reproduce Secondary Structures of ¡i¿De Novo¡/i¿ Designed Glu/Lys Peptides. *J. Chem. Theory Comput.* **2022**, *18*, 4070–4076.

(25) Ward, J. H. Hierarchical Grouping to Optimize an Objective Function. *J. Am. Stat. Assoc.* **1963**, *58*, 236–244.

(26) Ramos, J.; Lazaridis, T. Energetic Determinants of Oligomeric State Specificity in Coiled Coils. *JACS* **2006**, *128*, 15499–15510.

(27) Venkatakrishnan, A. J.; Fonseca, R.; Ma, A. K.; Hollingsworth, S. A.; Chemparathy, A.; Hilger, D.; Kooistra, A. J.; Ahmari, R.; Babu, M. M.; Kobilka, B. K.; Dror, R. O. Uncovering patterns of atomic interactions in static and dynamic structures of proteins. *bioRxiv* **2019**,

(28) Hedges, L. O.; Bariami, S.; Burman, M.; Clark, F.; Cossins, B. P.; Hardie, A.; Herz, A. M.; Lukauskis, D.; Mey, A. S. J. S.; Michel, J.; Scheen, J.; Suruzhon, M.; Woods, C. J.; Wu, Z. A Suite of Tutorials for the BioSimSpace Framework for Interoperable Biomolecular Simulation [Article v1.0]. *LiveCoMS* **2023**, *5*, 2375.

(29) Alibay, I.; Magarkar, A.; Seeliger, D.; Biggin, P. C. Evaluating the use of absolute binding free energy in the fragment optimisation process. *Commun. Chem.* **2022**, *5*, 105.

(30) Alibay, I. IAlibay/MDRestraintsGenerator: MDRestraintsGenerator v0.2.0. 2022; `https://doi.org/10.5281/zenodo.6972482`.

(31) Mobley, D. L.; Chodera, J. D.; Dill, K. A. On the use of orientational restraints and symmetry corrections in alchemical free energy calculations. *J. Chem. Phys.* **2006**, *125*, 084902.

(32) Fu, H.; Cai, W.; Hénin, J.; Roux, B.; Chipot, C. New Coarse Variables for the Accurate Determination of Standard Binding Free Energies. *J. Chem. Theory Comput.* **2017**, *13*, 5173–5178.

(33) Procacci, P.; Macchiagodena, M. On the NS-DSSB unidirectional estimates in the SAMPL6 SAMPLing challenge. *J. Comput. Aided Mol. Des.* **2021**, *35*, 1055–1065.

(34) Clark, F.; Robb, G.; Cole, D. J.; Michel, J. Comparison of Receptor–Ligand Restraint Schemes for Alchemical Absolute Binding Free Energy Calculations. *J. Chem. Theory Comput.* **2023**, *19*, 3686–3704.

(35) Heinzelmann, G.; Gilson, M. K. Automation of absolute protein-ligand binding free energy calculations for docking refinement and compound evaluation. *Sci. Rep.* **2021**, *11*, 1116.

(36) Fu, H.; HanatoK; Donyapour, N. fhh2626/BFEE2: BFEE 2.5.0. 2023; https://doi.org/10.5281/zenodo.10434156.

(37) Fu, H.; Chen, H.; Cai, W.; Shao, X.; Chipot, C. BFEE2: Automated, Streamlined, and Accurate Absolute Binding Free-Energy Calculations. *J. Chem. Inf. Model.* **2021**, *61*, 2116–2123.

(38) Fletcher, J. M.; Boyle, A. L.; Bruning, M.; Bartlett, G. J.; Vincent, T. L.; Zaccai, N. R.; Armstrong, C. T.; Bromley, E. H. C.; Booth, P. J.; Brady, R. L.; Thomson, A. R.; Woolfson, D. N. A Basis Set of *de Novo* Coiled-Coil Peptide Oligomers for Rational Protein Design and Synthetic Biology. *ACS Synth. Biol.* **2012**, *1*, 240–250.
